# A completeness-independent method for pre-selection of closely related genomes for species delineation in prokaryotes

**DOI:** 10.1186/s12864-020-6597-x

**Published:** 2020-02-26

**Authors:** Yizhuang Zhou, Jifang Zheng, Yepeng Wu, Wenting Zhang, Junfei Jin

**Affiliations:** 1 Laboratory of Hepatobiliary and Pancreatic Surgery, The Affiliated Hospital of Guilin Medical University, Guilin, Guangxi 541001 People’s Republic of China; 2 Peking-Tsinghua Center for Life Science, Academy for Advanced Interdisciplinary Studies, Peking University, Beijing, 100871 People’s Republic of China; 3 Guangxi Key Laboratory of Tumor Immunology and Microenvironmental Regulation, Guilin Medical University, Guilin, Guangxi 541001 People’s Republic of China; 4 China-USA Lipids in Health and Disease Research Center, Guilin Medical University, Guilin, Guangxi 541001 People’s Republic of China; 5 Guangxi Key Laboratory of Molecular Medicine in Liver Injury and Repair, Guilin Medical University, Guilin, Guangxi 541001 People’s Republic of China

**Keywords:** Tetranucleotide, Composition, Taxonomy, Species delineation, FRAGTE, Metagenomic binning, Average nucleotide identity

## Abstract

**Background:**

Whole-genome approaches are widely preferred for species delineation in prokaryotes. However, these methods require pairwise alignments and calculations at the whole-genome level and thus are computationally intensive. To address this problem, a strategy consisting of sieving (pre-selecting closely related genomes) followed by alignment and calculation has been proposed.

**Results:**

Here, we initially test a published approach called “genome-wide tetranucleotide frequency correlation coefficient” (TETRA), which is specially tailored for sieving. Our results show that sieving by TETRA requires > 40% completeness for both genomes of a pair to yield > 95% sensitivity, indicating that TETRA is completeness-dependent. Accordingly, we develop a novel algorithm called “fragment tetranucleotide frequency correlation coefficient” (FRAGTE), which uses fragments rather than whole genomes for sieving. Our results show that FRAGTE achieves ~ 100% sensitivity and high specificity on simulated genomes, real genomes and metagenome-assembled genomes, demonstrating that FRAGTE is completeness-independent. Additionally, FRAGTE sieved a reduced number of total genomes for subsequent alignment and calculation to greatly improve computational efficiency for the process after sieving. Aside from this computational improvement, FRAGTE also reduces the computational cost for the sieving process. Consequently, FRAGTE extremely improves run efficiency for both the processes of sieving and after sieving (subsequent alignment and calculation) to together accelerate genome-wide species delineation.

**Conclusions:**

FRAGTE is a completeness-independent algorithm for sieving. Due to its high sensitivity, high specificity, highly reduced number of sieved genomes and highly improved runtime, FRAGTE will be helpful for whole-genome approaches to facilitate taxonomic studies in prokaryotes.

## Background

Species delineation among prokaryotes is harder and more controversial than among eukaryotes [1], mainly due to the lack of species concepts [2, 3]. Historically, microbial species delineation has not been driven by theory-based concepts [3], but progressed through a series of empirical improvements in parallel with technical developments instead [1]. Recent advances in sequencing technologies have brought species delineation into the genomic era. A widely-used approach is the Average Nucleotide Identity (ANI), which computationally mimics DNA-DNA hybridization through overcoming its shortcomings including experimental complexity, labor-intensive operation and non-incremental results [4–7]. Other such approaches include the average amino-acid identity [8, 9] and the Microbial Species Identifier (MiSI) [10]. All these approaches are based on whole genomes and thus have higher resolution and more accurate and reliable than gene-based approaches, including those based on a single gene such as 16S rRNA [11] or those based on several housekeeping genes such as the species identification tool [12], multilocus sequence typing [13] and multilocus sequence analysis [14].

However, genome-based approaches are based on computationally intensive pairwise genomic alignments and calculations, which are a disadvantage in large-scale studies against many reference genomes. For example, a comparison of 10,000 genomes against 1000 genome references results in 10,000,000 alignment pairs. In reality, the National Center for Biotechnology Information (NCBI) database contains 83,075 genomes belonging to 19,190 putative species (up to 20 January 2017), though this is likely to rise sharply in the face of the increasing rate of species discovering and strain sequencing. Thus, the development of new approaches with improved computational efficiency is crucial.

In theory, genome-based species delineation requires alignment and calculation of intraspecies strains only. However, a number of interspecies strains are inevitably compared. A strategy to reduce computing cost would be through “sieving”, which is selecting closely related (intraspecies and some closely related interspecies) pairs from total pairs before alignment and calculation (Additional file 1: Figure S1). Under this strategy, species delineation consists of the sieving process followed by the process of alignment and calculation. It is important to emphasize that sieving does not directly delineate species, as some interspecies pairs are still able to perforate the mesh of the sieving algorithm. Therefore, sieving is not a substitute for genome-based approaches such as the ANI approach.

**Fig. 1:**
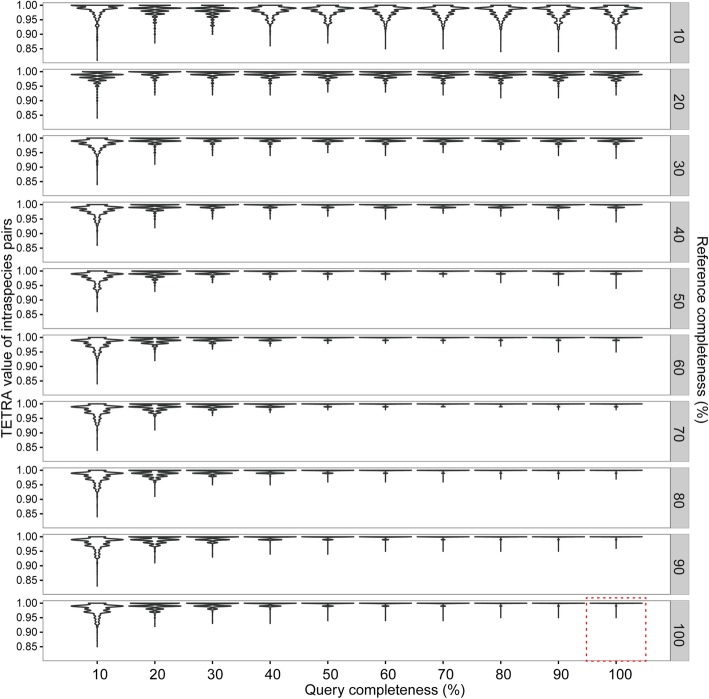
Impact of genomic completeness on TETRA values. Each plot row shows a different level of completeness for the reference genomes. Dashed box, queries with 100% completeness versus references with 100% completeness. All were run on 1779 queries (Additional file 2: Table S1) against 264 references (Additional file 2: Table S2) with 10–100% of genome completeness

**Fig. 2:**
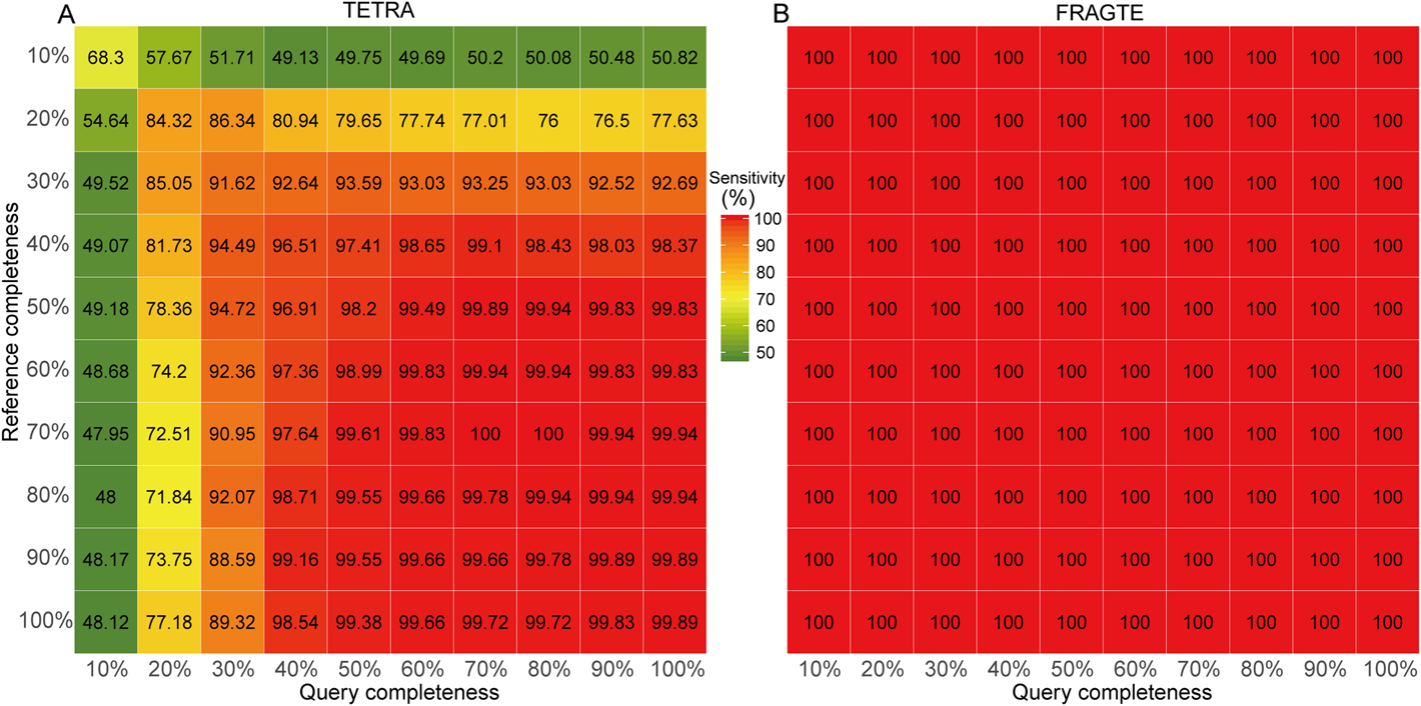
Sieving sensitivity of the TETRA and FRAGTE approaches on simulated genomes. **a** for TETRA; **b** for FRAGTE. All were run on the 1779 queries against 264 references with 10–100% of genome completeness,. The number in cell is sensitivity (%), which is calculated using the number of sieved intraspecies pairs divided by 1779 and used as a basis for color intensity

Genomic composition is species specific [15–19] and can be used to indicate relationships among species. The ability to distinguish genomic composition goes up with oligonucleotide sizes [19–22]. However, the computing cost also correspondingly increases. For compromise between the distinguishing power and the computing cost, tetranucleotide is widely used [23–28]. Before our published method called“Tetranucleotide-derived Z-value Manhattan Distance” (TZMD), a total of four statistical methods have been published for tetranucleotide profiling, including the zero-order Markov method [22, 25], the maximal-order Markov method [22, 25], the “relative tetranucleotide frequency” method [16] and the z-value method [19]. All of these methods uses the Pearson correlation coefficient distance (PCCD) to assess composition similarity between two genomes [19, 22]. Our previous study showed that the approach using PCCD for genome-wide **TETRA**nucleotide z-value (TETRA) is able to represent the three other statistical methods [29]. In addition, TETRA is alignment-free and accordingly requires less computing cost than alignment-based approaches. Although Richter et al. [4] developed the TETRA approach to sieve pairs for species delineation, our previous study showed that TETRA is affected by genomic completeness [29]. In this study, we also found that TETRA is completeness-dependent (Fig. 1) and is not suitable for incomplete genomes, especially those with < 40% completeness (Fig. 2a). As TZMD is more susceptible to genome incompleteness than TETRA [29], we just used TETRA as the reference method for comparion in this study.

Here, we developed a completeness-independent method termed **FRAG**ment **TE**trinucleotide frequency PCCD (FRAGTE). Our results showed that FRAGTE dramatically improves sieving sensitivity (the number of sieved intraspecies pairs divided by the total number of intraspecies pairs) as well as sieving specificity (the number of correctly filtered interspecies pairs divided by the total number of interspecies pairs). Additionally, FRAGTE reduces the number of totally sieved pairs (including intra- and some inter-species pairs) to greatly lower the required computing cost for subsequent alignment and calculation. Also, we showed that FRAGTE runs faster than TETRA. Thus, FRAGTE will assist all genome-based species-delineation approaches to facilitate taxonomic studies for prokaryotes in the future.

## Results

### Sieving by TETRA depends on genome completeness

Genome-based approaches require pairwise genome-wide alignments. To reduce the computing cost, Richter *et al.* [4] developed an alignment-free TETRA approach to retrieve or “sieve” only closely related pairs for subsequent alignment and ANI calculation. These authors calculated the TETRA values fully according to Teeling *et al.* algorithm [19]. In brief, TETRA first counts the observed tetranucleotide frequencies, as well as trinucleotide and dinucleotide frequencies. Then, it calculates the expected tetranucleotide frequencies using a maximal-order Markov model. Subsequently, it measures the divergence between observed and expected frequencies as z-scores with additional consideration of variances. Finally, it assesses composition similarity between a pair of genomes by calculating the PCCD for their z-scores. These authors found that TETRA values correlated strongly with ANI values in high ANI value zone (Fig. 3 of [4]) and most intraspecies TETRA values were > 0.99 [4]. Therefore, 0.99 was recommended as the TETRA criterion to sieve closely related genomes. In this way, TETRA greatly decreases the amount of pairs required for alignment and ANI calculation, which considerably improves computation efficiency. It is also worth pointing out that TETRA also sieves some closely related interspecies genomes with similar composition (Additional file 1: Figure S1) and thus TETRA might not be used to delineate species directly but just to sieve closely related genomes for subsequent species delineation [4]. However, our results showed that genome completeness strongly affected TETRA values (Fig. 1). As expected, genome completeness affected sieving especially for genomes with completeness < 40% to sieve only < 95% of intraspecies genomes (Fig. 2a), showing that TETRA is completeness-dependent.

**Fig. 3:**
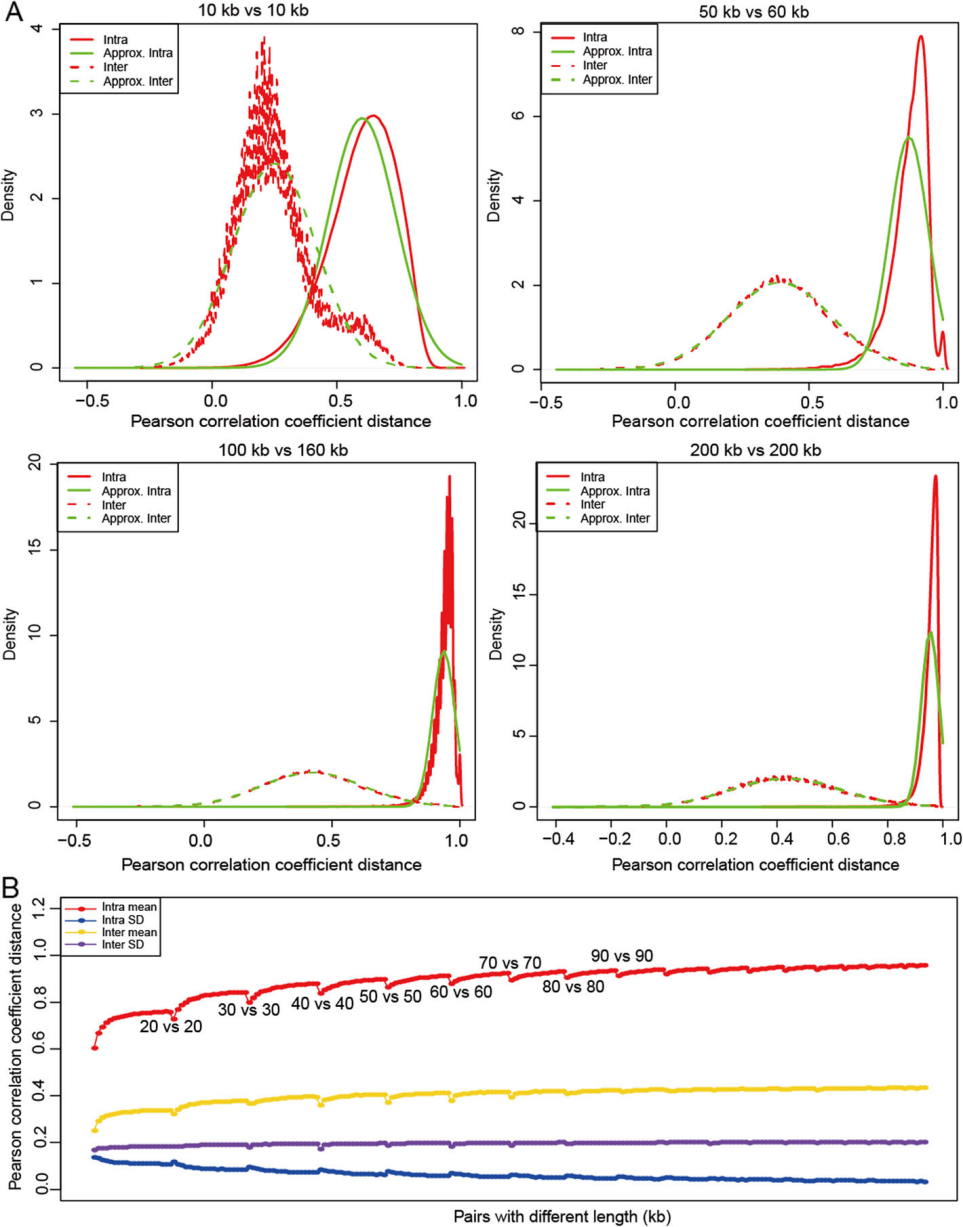
Distribution of inter- and intra-species Pearson correlation coefficient distances (PCCDs). **a** Four selected examples with different sizes are presented. Inter- and intra-species PCCDs can be approximated by normal distributions (*P*-values < 2.2e-16, one-sample Kolmogorov–Smirnov test). **b** Mean and standard deviation (SD) of intra- and inter-species PCCD distribution. For pairwise sizes in the x axis, please refer to Additional file 3

Although most TETRA values for complete genomes are ≥0.99, there are two exceptions (Fig. 1), including the value of 0.95 for *Borreliella burgdorferi* strains CA382 and B31, and the value of 0.97 for *Borrelia hermsii* strains HS1 and CC1. Our checking found that the two varied compositions were both attributed to their plasmid differences (Additional file 1: Figure S2). This demonstrates that TETRA is not the ideal method to detect all intraspecies genomes even when these genomes are complete. Also, this indicates that composition is genome specific, requiring developing a genome-specific cutoff (GSC) to reflect the genome-specific feature of composition.

### Empirical analysis of fragment intra- versus (vs.) inter-species PCCD distributions

It has been reported that intragenomic differences are generally smaller than intergenomic differences in genomic composition [15–19]. This feature is widely used for metagenomic binning (classifying metagenomic assemblies into species-specific groups) [30–33], implying that fragments are able to indicate species relationship and can be used to select closely related genomes. Thus, we devised an approach based on fragment rather than whole genome to overcome the above limitation of TETRA. To use fragments, we used 2043 complete genomes with unambiguous species affiliations including 1779 queries (Additional file 2: Table S1) and 264 references (Additional file 2: Table S2) to summarize the information useful for designing FRAGTE.

Our results showed that the intra- vs. inter-species PCCD distributions of long fragments (> 10 kilobase pair, kb) were well separated (Fig. 3a), but not those of short fragments (data not shown). Therefore, one possible advantage of using fragment rather than whole genome is completeness-independent, only requiring fragments with length > 10 kb. To further assess the effect of fragment size on PCCD, we tested pairs ranging from 10 kb to 200 kb in length. Our empirical analysis showed that each intra- or inter-species PCCD distribution was approximated by a normal distribution (*P*-value < 2.2e-16, one-sample Kolmogorov–Smirnov test) (Fig. 3a). Additionally, our results showed that the average intraspecies PCCDs increased with fragment size, while their standard deviations (SDs) correspondingly decreased (Fig. 3b and Additional file 3). In contrast, both the average and SD of interspecies PCCDs increased slightly. These results imply that the ability to distinguish species increases with fragment size and thus a unified cutoff cannot be set to differentiate species, supporting the idea that setting a rigid cutoff of 0.99 in TETRA is not appropriate.

As we determined the intra- vs. inter-species PCCD distributions for pairs with lengths ranging from 10 kb to 200 kb, we were able to determine the length-specific cutoffs (LSCs) to decide which genomes were closely related. Here, we determined two cutoffs for fragments with a pair of given lengths: one cutoff to include at least 95% of intraspecies pairs based on the above-determined intra-species PCCD distribution and the other cutoff to exclude at least of 95% of interspecies pairs based on the above-determined inter-species PCCD distribution. The smaller cutoff was chosen as the LSC (Additional file 1: Figure S3A), to ensure that almost 100% of intraspecies pairs were selected. Our assessment from the above-determined PCCD distributions (Additional file 3) showed that the LSCs for large-sized fragments (> 60 kb) achieved > 99.87% of sensitivity (Additional file 1: Figure S3B), while the LSCs for small-sized fragments (< 60 kb) showed considerably less sensitivity (Additional file 1: Figure S3B and S3C). Therefore, we designed an elaborate strategy in FRAGTE to improve sensitivity for small-sized fragments as described in the next section (Additional file 1: Figure S3C).

### Algorithm description

The FRAGTE approach was designed to use fragments rather than whole genomes. To use LSCs, FRAGTE divides each genome into fragments and selects a typical fragment to represent that genome. Besides, composition is genome-specific, as indicated by the two exceptions (Fig. 1), possibly due to (but not limited to) plasmid differences (Additional file 1: Figure S2). However, LSCs were drawn from empirically determined PCCD distributions (Fig. 3) and were not genome-specific. As a genome can be divided into multiple fragments, a GSC can be calculated as the mean intragenomic PCCD minus two SDs based on all its divided fragments with two additional restrictions (for details, see Materials and Methods). Taking 1779 queries with 60% genome completeness as an example, we found that their GSCs broadly ranged from 0.75 to 0.92, efficiently reflecting the individuality of each genome (Additional file 1: Figure S4). Therefore, we designed FRAGTE to use LSCs for genome selecting and then GSCs for genome filtering to ensure both high sensitivity and high specificity.

**Fig. 4:**
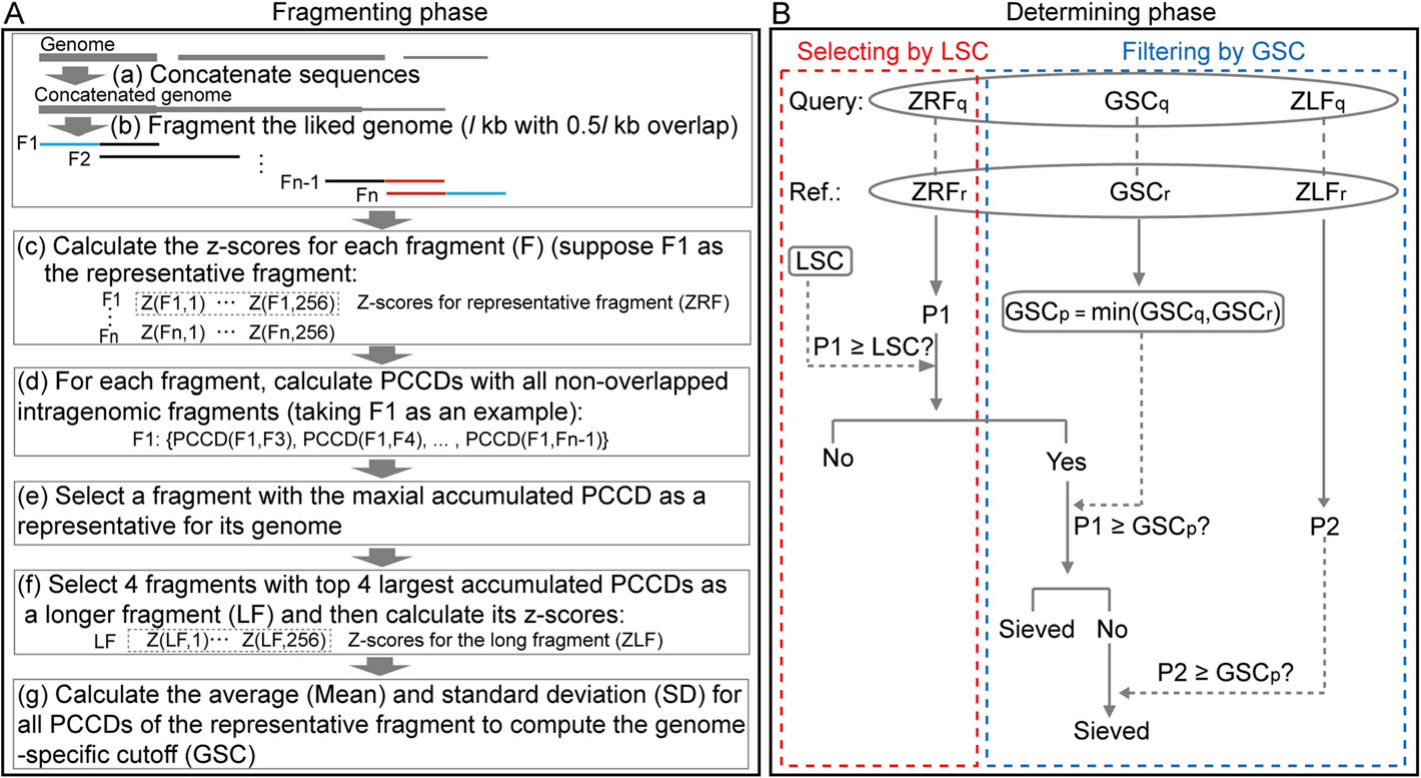
Outline of the FRAGTE approach. A, fragmenting phase. An incomplete genome is concatenated (**a**). Then the concatenated genome is divided by a sliding *l-*kb window with 0.5 *l*-kb overlap (**b**) and 256 z-scores are calculated for each fragment (**c**). For each fragment, PCCDs are calculated with all non-overlapped intragenomic fragments (**d**) and then summed as an accumulated PCCD. Subsequently, a representative fragment with the maximal accumulated PCCD is determined for its genome (**e**) and its z-scores is selected as z-scores for representative fragment (ZRF). Besides, 4 fragments with top 4 largest accumulated PCCDs are used to calculate z-scores for long fragment (ZLF) (**f**). Finally, the average PCCD and standard deviation (SD) based on all PCCDs of the representative fragment are calculated and genome-specific cutoff (GSC) is thus computed as the mean intragenomic PCCD minus two SDs with two restrictions (**g**). In this way, FRAGTE finishes fragmenting phase and obtains z-scores for the representative fragment (ZRF) and the fourfold longer fragment (ZLF), as well as a GSC. **b** determining phase. a PCCD (P1) based on ZRFs is calculated. If P1 > LSC, the pair is selected. To improve specificity, GSC is used. GSC for a pair (GSC_p_) is determined as the smaller between GSC for the query (GSC_q_) and for the reference (GSC_r_). If P1 > GSC_p_, this pair is finally sieved. Otherwise, a second PCCD (P2) based on ZLFs is calculated. If P2 > GSC_p_, this pair is sieved

FRAGTE consists of fragmenting phase followed by determining phase. In the fragmenting phase, it divides each genome into fragments and then selects a representative fragment. If an incomplete genome has multiple contigs/scaffolds, FRAGTE first concatenates contigs/scaffolds for this genome (Fig. 4a). Subsequently, FRAGTE divides the (concatenated) genome by a sliding window of *l* kb (with 0.5 *l* kb overlap). Here, we devised FRAGTE to divide each genome into fragments as long as possible (Additional file 1: Figure S5, for details, see Materials and Methods), considering the two following benefits. One is to increase selecting sensitivity by LSC, as selecting sensitivity by LSC increases with fragment size (Additional file 1: Figure S3B). The other is to increase filtering power by GSC, as the average intraspecies PCCD increases and the SD of intraspecies PCCDs decreases with fragment size (Fig. 3b) to yield a large GSC. Then, FRAGTE calculates 256 z-scores for all fragments as described in Teeling et al. [19].

Next, for each fragment, FRAGTE calculates PCCDs with all non-overlapped intragenomic fragments. In this way, a set of PCCDs is obtained for each fragment. FRAGTE calculates the accumulated PCCD for each fragment by summing all its PCCDs. Then, FRAGTE selects the fragment with the largest accumulated PCCD to represent its genome and obtains z-scores for the representative fragment (ZRF). As FRAGTE divides a genome into fragments as long as possible, it may filter some intraspecies pairs due to the large GSCs derived from increased average but decreased SD of intraspecies PCCDs from long fragments. To improve on this, FRAGTE was made to use an even longer fragment (concatenated from 4 fragments with top 4 largest accumulated PCCDs) to possibly yield a larger PCCD than that normally obtained from shorter fragment (the divided fragment) for a given intraspecies pair. Fortunately, the average interspecies PCCDs only increase slightly (Fig. 3b), implying that using a longer fragment does not greatly increase the amount of sieved interspecies pairs to keep its high specificity. In this context, FRAGTE additionally uses the fourfold longer fragment to generate a PCCD for comparing with the GSC calculated from the divided fragments. Using this strategy, FRAGTE is able to ensure both high specificity and high sensitivity. Thus, FRAGTE additonally selects 4 fragments with top 4 largest accumulated PCCDs to form a fourfold longer fragment and calculated z-scores for the fourfold longer fragment (ZLF) as described in Teeling et al. [19]. Besides, as LSCs affect the sensitivity of small-sized pairs (< 60 kb) and their selecting sensitivities increase with fragment size (Additional file 1: Figure S3B), we designed FRAGTE to use the fourfold longer fragment (i.e. ZLF) instead of its representative fragment (i.e. ZRF) for selecting by LSC, when the size of the fourfold longer fragment is ≤200 kb. By this means, the selecting sensitivity is dramatically improved to ensure that almost 100% of intraspecies pairs are selected by LSC (Additional file 1: Figure S3C). Finally, FRAGTE calculates both mean and SD for all PCCDs of the representative fragment to compute a GSC (for details, see Materials and Methods). Up to this step, FRAGTE finishes all intragenomic processing in the fragmenting phase.

In the determining phase of FRAGTE, it effectively assesses if genomes are closely related. It calculates an intergenomic PCCD (termed P1) between a pair of genomes based on their ZRFs (Fig. 4b). If the P1 is larger than its LSC, this pair is considered to be the same species. To further improve specificity, GSCs are then used to filter pairs. For a given pair, two GSCs are obtained and the smaller one is taken as the GSC for this pair (term GSC_p_). Determining GSC_p_ by this means has two benefits. One is automatically determining the cutoff, without setting a prior cutoff as in TETRA. The other is that the cutoff is genome-specific (Additional file 1: Figure S4), unlike TETRA using a rigid cutoff of 0.99. If the P1 is larger than its GSC_p_, the pair is considered to be closely related and thus sieved. Otherwise, FRAGTE calculates a second PCCD (termed P2) based on their ZLFs. If the P2 is larger than its GSC_p_, this pair is considered to be closely related and consequently sieved.

### Sieving performance on simulated genomes

**Fig. 5:**
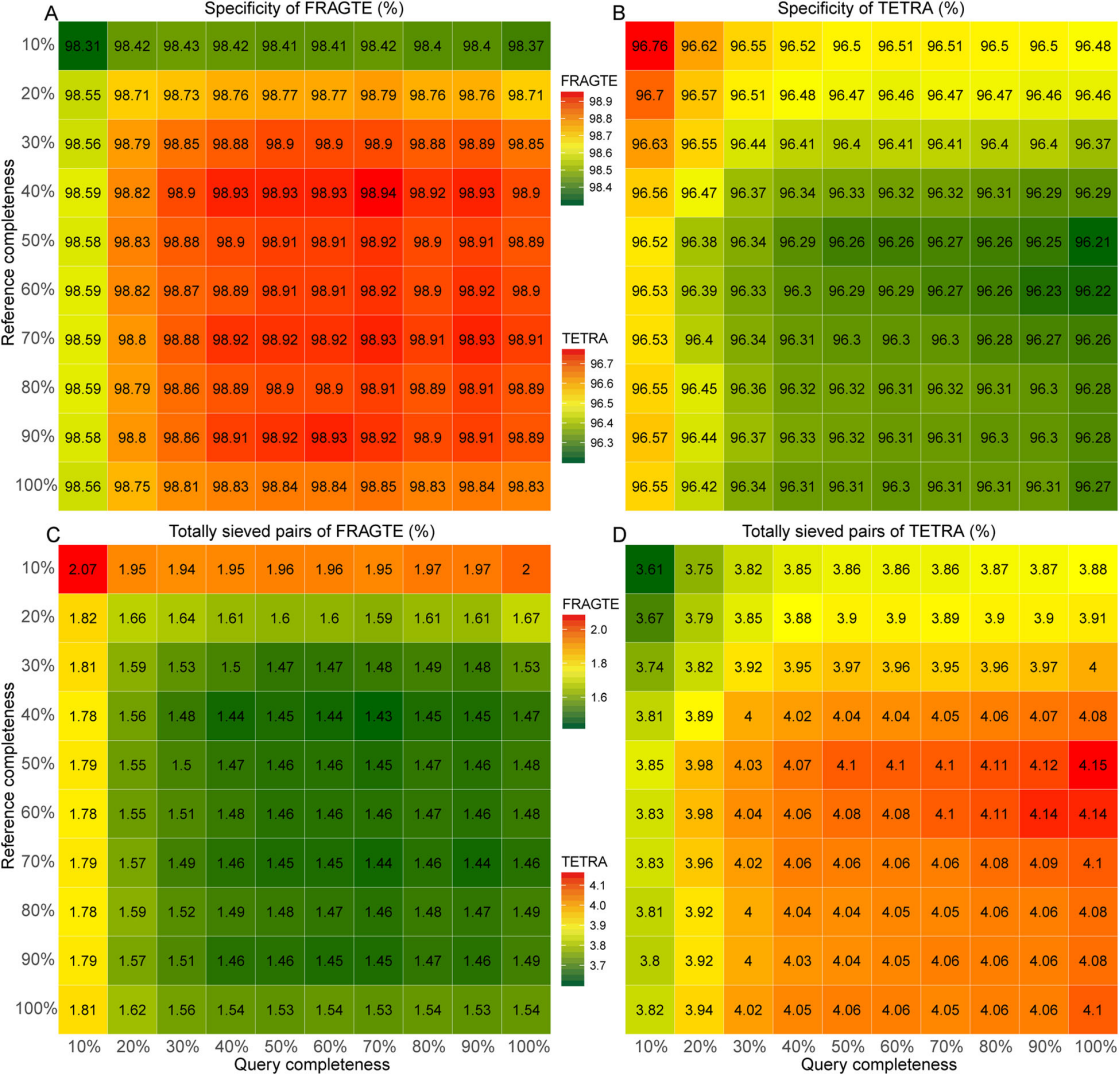
Specificity and percentage of totally selected pairs for the FRAGTE and TETRA approaches on simulated genomes. **a** for specificity of FRAGTE; **b** for specificity of TETRA; **c** for totally sieved pairs of FRAGTE; **d** for totally sieved pairs of TETRA. All were run on the 1779 queries against 264 references with 10–100% of genome completeness, totally comprising 469,656 pairs. The specificity (%) in cell is calculated using the number of correctly filtered interspecies pairs divided by the total number of interspecies pairs. The percentage of totally sieved pairs in cell is calculated using the total number of sieved pairs divided by the total number of pairs. The number in cell is used as a basis for color intensity

All 1779 query (Additional file 2: Table S1) and 264 reference genomes (Additional file 2: Table S2) with unambiguous species relationships (> 96% ANI) were selected to investigate the sieving performance of the FRAGTE approach. We extracted 10–100% of genomes to assess the effect of completeness on the sieving performance. Our results showed that FRAGTE strikingly yielded perfect sensitivities of 100%, regardless of their completeness (Fig. 2b). Compared with TETRA, FRAGTE achieved sensitivity with ~ 50% improvement for the genomes with 10% completeness and even slight improvement for the complete genomes (Fig. 2). Besides, FRAGTE correctly filtered about 98.31–98.94% of interspecies pairs (Fig. 5a), while TETRA only correctly filtered about 96.21–96.76% interspecies pairs to achieve the same sensitivities as FRAGTE (Fig. 5b), demonstrating that FRAGTE has higher specificity than TETRA. Collectively, FRAGTE achieved both high sensitivity and high specificity. Due to its high specificity, FRAGTE sieved only approximately 1.43–2.07% of total pairs (including intra- and some inter-species pairs) to achieve its 100% of sensitivities (Fig. 5c). Then, we compared the total number of sieved pairs of FRAGTE with that of TETRA when TETRA achieved the same sensitivities as FRAGTE. Our results showed that the total number of sieved pairs by FRAGTE were ~ 42.66% to ~ 64.79% lower than those by TETRA (Fig. 5c and d), demonstrating that FRAGTE greatly reduces the amount of pairs for subsequent alignment and calculation and thus greatly reduces computing cost.

**Fig. 6:**
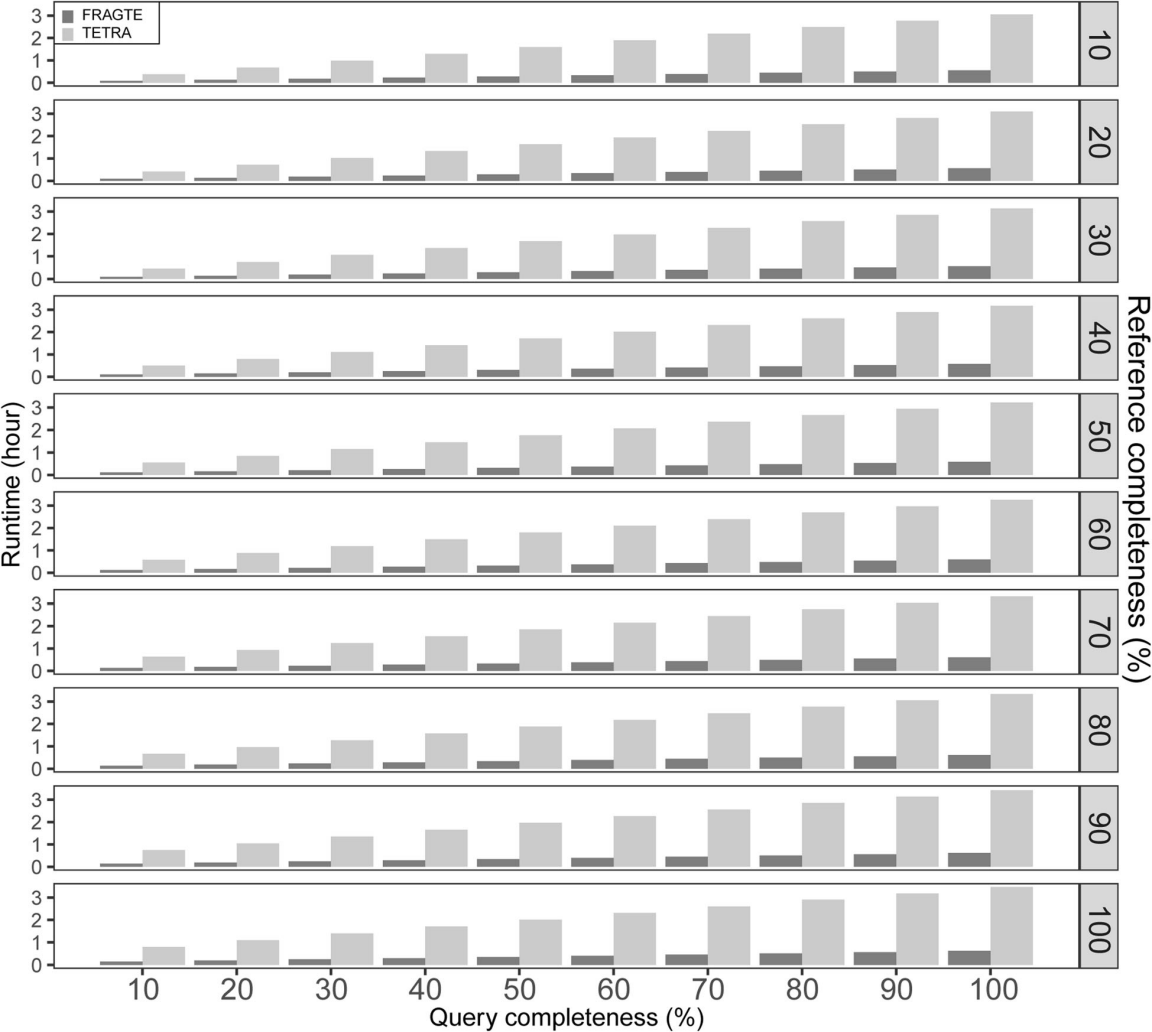
Runtime comparison of FRAGTE and TETRA on simulated genomes. All were run on the 1779 queries against 264 references with 10–100% of genome completeness. The runtime is the summed executive time including both fragmenting and determining phrases for all 469,656 pairs by using serial execution (single thread, single process). Only a single compute node with two Intel® Xeon® Silver 4114 20-core processors was used

Although FRAGTE highly reduced the computing cost for species delineation through sieving a lower number of pairs in total for subsequent alignment and calculation than TETRA, we tested whether FRAGTE itself reduced runtime of the process sieving. For this, we used a single compute node with two Intel® Xeon® Silver 4114 20-core processors and evaluated the runtime of both FRAGTE and TETRA using serial execution (single thread, single process). We observed that FRAGTE drastically reduced ~ 77–82% of runtime of TETRA (Fig. 6). Therefore, FRAGTE extremely reduced the executive time for the process of sieving as well as the process of alignment and calculation after sieving due to reduced amount of all sieved pairs, which together improved the run efficiency for genome-wide species delineation. In conclusion, all these findings demonstrated that FRAGTE performs better than TETRA for sieving.

### Sieving performance on real genomes

**Fig. 7:**
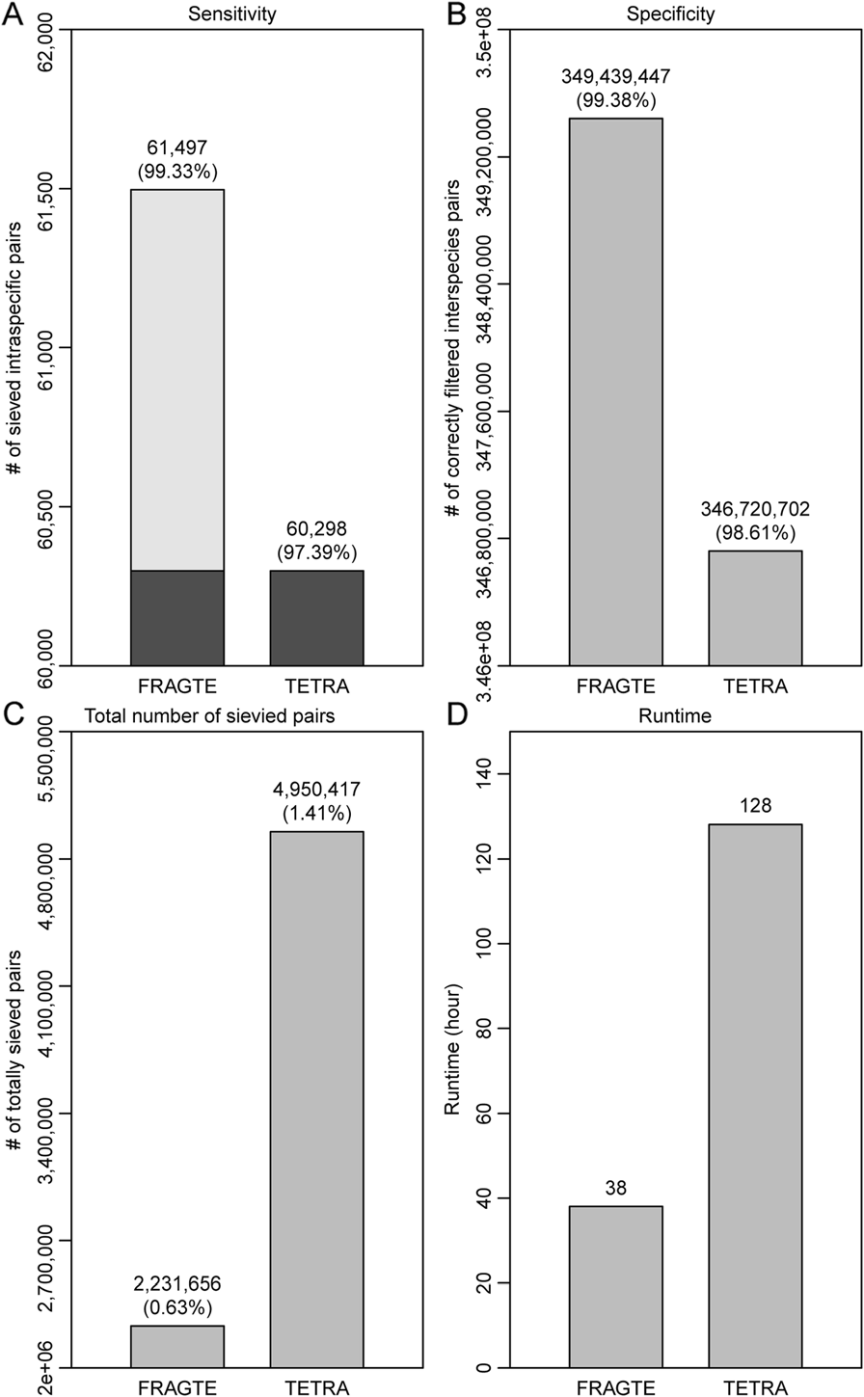
Sieving performance on real genomes. **a** for sensitivity. Black, 60,298 intraspecies pairs sieved by both FRAGTE and TETRA; gray, 1199 intraspecies pairs uniquely detected by FRAGTE. **b** for specificity. **c** for total number of sievied pairs. **d** for runtime. The runtime is the summed executive time including both fragmenting and determining phrases for all pairs by using serial execution (single thread, single process). Only a single compute node with two Intel® Xeon® Silver 4114 20-core processors was used. All were run on 61,914 queries (Additional file 2: Table S3) against 5680 references (Additional file 2: Table S4), which totally comprise 351,671,520 pairs

Artificial simulation may generate non-overlapping regions for intraspecies pairs. In contrast, real genomes for different intraspecies strains contain overlapping (aligned) regions, possibly because aligned regions have similar features such as G + C features and thus are similarly readily to assemble. Accordingly, sieving using simulation data cannot completely reflect the sieving performance on the real data. Consequently, we assessed the sieving performance on the real genomes. For this, 61,914 query (Additional file 2: Table S3) and 5680 reference genomes (Additional file 2: Table S4), which comprised 61,914 labeled intraspecies pairs and 351,609,606 interspecies pairs, were used. Our checking found that 58,120 (93.87%) of queries and 4335 (76.32%) of references were incomplete and thus were suitable to assess the impact of completeness on the sieving performance of both the FRAGTE and TETRA approaches. FRAGTE sieved 61,497 (99.33%) intraspecies pairs, while TETRA sieved 60,298 (97.48%) using its criterion of 0.99 (Fig. 7a), demonstrating that FRAGTE is more sensitive. Strikingly, FRAGTE detected all 60,298 intraspecies pairs sieved by TETRA (Fig. 7a), implying that FRAGTE can completely substitute TETRA. To find out why 417 pairs labeled as intraspecies in the NCBI taxonomy database were not successfully sieved, we calculated their ANIs and alignment fractions (AFs) by using the MUMmer algorithm (version 3.23) according to the method in [4]. We found that only 15 of them had an ANI > 96% and an AF > 60%. Therefore, according to the MiSI method [10], from these 417 pairs, only 15 pairs were truly intraspecies. Thus, FRAGTE substantially achieved a nearly perfect sensitivity of about 99.98%, which was similar to the sensitivity obtained with the simulated genomes (Fig. 2). Also, we found that FRAGTE correctly filtered more interspecies pairs than TETRA when TETRA achieved the same sensitivity as FRAGTE (Fig. 7b), supporting that FRAGTE is more specific than TETRA for sieving. Taken together, FRAGTE achieved both higher sensitivity and higher specificity on real genomes than that by the TETRA approach, demonstrating that FRAGTE is also greatly useful for practical/real genomes. In addition, our analysis showed that FRAGTE sieved a total of 2,231,656 (0.63%) pairs, while TETRA sieved a total of 4,950,417 (1.41%) pairs to achieve the same sensitivity as FRAGTE (Fig. 7c), showing that FRAGTE dramatically reduced the required computing cost for subsequent species delineation. Finally, we used a single compute node with two Intel® Xeon® Silver 4114 20-core processors as well as serial execution to compare the runtime of FRAGTE with that of TETRA. We found that FRAGTE amazingly reduced the execution time to approximatively 29.69% of that of TETRA (Fig. 7d). Consequently, we proved that FRAGTE extremely improved run efficiency for the process of sieving as well as subsequent alignment and calculation to together accelerate genome-wide species delineation.

### Sieving performance on metagenome-assembled genomes (MAGs)

**Fig. 8:**
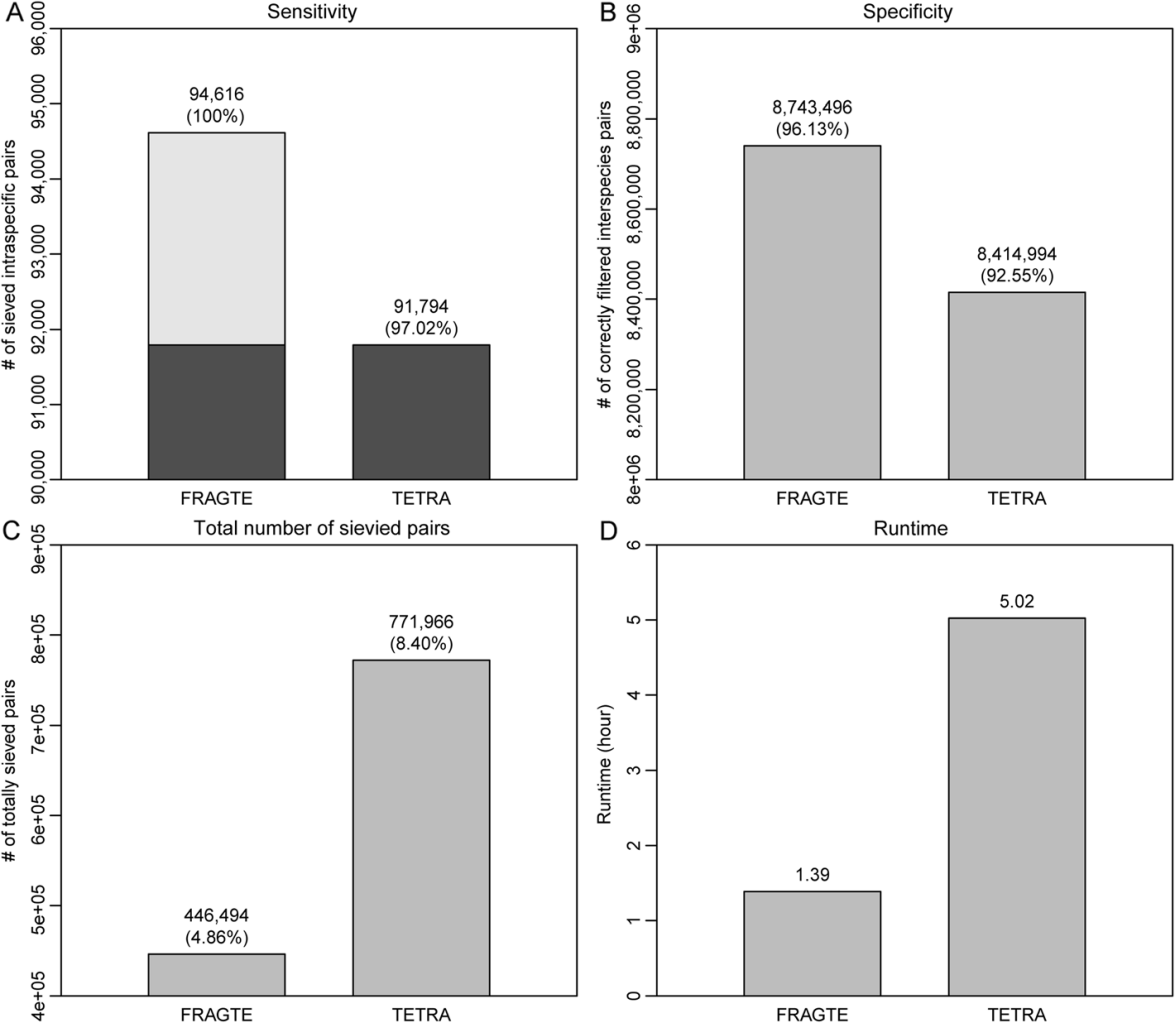
Sieving performance on metagenome-assembled genomes (MAGs). **a** for sensitivity. Black, 91,794 intraspecies pairs sieved by both FRAGTE and TETRA; gray, 2822 intraspecies pairs uniquely detected by FRAGTE. **b** for specificity. **c** for total number of sievied pairs. **d** for runtime. The runtime is the summed executive time including both fragmenting and determining phrases for all pairs by using serial execution (single thread, single process). Only a single compute node with two Intel® Xeon® Silver 4114 20-core processors was used. All were run on 3032 MAGs (Additional file 2: Table S5) against themselves, which comprise 94,618 intra- and 9,095,374 inter-species pairs

MAGs are genomes reconstructed from the metagenomic data (microbial communities). However, due to the complexity of metagenome, MAGs may be more fragmented than genomes recovered from isolates, manifested by the larger numbers of their contigs/scaffolds (Additional file 1: Figure S6A) or smaller sizes of their contigs/scaffolds (Additional file 1: Figure S6B). Accordingly, sieving using both the above simulated and real genomes cannot completely reflect the sieving performance on MAGs. Consequently, we assessed the sieving performance on MAGs. For this, 3032 MAGs (Additional file 2: Table S5) against themselves, which comprised 94,618 intra- and 9,095,374 inter-species pairs, were used. FRAGTE sieved 94,616 intraspecies pairs to achieve a nearly perfect sensitivity of ~ 100% (Fig. 8a), which was similar to the sensitivity obtained with the simulated genomes (Fig. 2b) or the real genomes (Fig. 7a). In contrast, TETRA sieved 91,794 (97.02%) intraspecies pairs, supporting that FRAGTE is more sensitive. Surprisingly, FRAGTE detected all 91,794 intraspecies pairs sieved by TETRA (Fig. 8a), indicating that FRAGTE can completely substitute TETRA. Meanwhile, FRAGTE correctly filtered 8,743,496 (96.13%) interspecies pairs, while TETRA only correctly filtered 8,414,994 (92.55%) interspecies pairs to achieve the same sensitivity as FRAGTE (Fig. 8b), demonstrating that FRAGTE has higher specificity than TETRA. Collectively, FRAGTE achieved both higher sensitivity and higher specificity on MAGs than that by the TETRA approach, demonstrating that FRAGTE is also greatly useful for MAGs.

Due to the improved sieving specificity, FRAGTE sieved only a total of 446,494 (4.86%) pairs, while TETRA sieved a increased number of 771,966 (8.40%) pairs to achieve the same sensitivity as FRAGTE (Fig. 8c). This result indicates that FRAGTE dramatically reduces the required computing cost for subsequent alignment and calculation. Additionally, we compared the runtime of FRAGTE with that of TETRA by using a single compute node with two Intel® Xeon® Silver 4114 20-core processors and serial execution. We found that FRAGTE amazingly reduced the execution time to approximatively 27.69% of that of TETRA (Fig. 8d). Consequently, we proved that FRAGTE extremely improves run efficiency for both the processes of sieving and after sieving (subsequent alignment and calculation) to together accelerate genome-wide species delineation.

## Discussion

### The need for a completeness-independent sieving approach

Although we are able to obtain complete or near-complete genomes more readily than before, due to recently emerging long-read sequencing technologies such as single-molecule, real-time (SMRT) and Oxford Nanopore MinION sequencing [34–37], it is still difficult to obtain high-completeness genomes for uncultured microbes. The genomes of uncultured organisms, which cannot be obtained by traditionally sequencing monocultures, are alternatively obtained via binning metagenomic assemblies or single-cell sequencing. Genomes binned from metagenomic assemblies are mostly drafts [38–40], due to the complex nature of metagenomes. Although SMRT sequencing is also able to improve assembly to yield even complete genomes, it is still challenging to generate finished genomes for most such microbes [41]. In addition, SMRT sequencing requires a high biomass for library preparation, hindering its ability to generate complete genomes for low-biomass microbiomes like those of the skin. Additionally, the Hierarchical Genome Assembly Process, an assembler for SMRT data *in solo*, has inherent technical limitations, requiring high sequence coverage and read overlap for consensus calling and preassembly [34], making it difficult to generate finished genomes. Similarly, single-cell genomics usually generates draft genomes [42, 43] due to its intrinsic technological challenges including cell isolation, chimeric DNA-molecules, and amplification bias [44, 45]. Besides, the majority of available genomes in public databases are unfinished. In the NCBI database, only 7.5% (6230/83075) of the genomes are complete. Therefore, developing a completeness-independent approach like FRAGTE is indispensable.

### Comparison of FRAGTE and FastANI

Similar to TETRA, FastANI is also an alignment-free approach. It estimates ANI based on Jaccard similarity of genomic k-mers to greatly speed up the calculation of ANI [46]. Accordingly, FastANI is not directly comparable with FRAGTE, as FastANI is just another variation of the ANI calculation algorithm, not tailored specially for sieving according to Additional file 1: Figure S1. One another reason why FRAGTE is incomparable with FastANI is that FastANI acheives lower sensitivities than FRAGTE in some condtions. Taking the 3032 MAGs as an example, we run FastANI with its default parameter settings and found that FastANI sieved only about 22.80% of intraspecies pairs (Additional file 1: Figure S7A), indicating that FastANI is also completeness-dependent. Thus, we cannot compare FastANI with FRAGTE in terms of specificity, total number of sieved pairs and runtime (Additional file 1: Figure S7B-D), as they sievied different numbers of intraspecies pairs. However, we still compared FRAGTE with FastANI for the 1779 queries against 264 references. Our results showed that FastANI sieved 100% of intraspecies pairs only when at least one genome of a pair is > 30% complete (Additional file 1: Figure S8A), again indicating that FastANI is completeness-dependent. Then, we compared their specificities, total numbers of sieved pairs and execution times when FastANI achieved the same sensitivities as FRAGTE. We found that FRAGTE correctly filtered more interspecies pairs than FastANI (Additional file 1: Figure S8B and Fig. 5a), showing that FRAGTE achieves higher specificity than FastANI. With respect to total number of sieved pairs, our results showed that FastANI sieved a larger total number of pairs than FRAGTE (Additional file 1: Figure S8C and Fig. 5c). Regarding runtime, we found that FRAGTE run faster than FastANI (Additional file 1: Figure S8D and Fig. 6). Overall, FRAGTE is superior to FastANI as the sieving algorithm to decide closely related genomes for species delineation.

### Comparison of FRAGTE and alignment-based approaches

Genome-wide delineation approaches require pairwise alignment and calculation at the genome level. In theory, alignment-based approaches on the basis of a single or several marker genes can also be used for sieving, as they greatly reduce the computing cost from the genome level to the gene level. In principle, any housekeeping gene such as 16S rRNA, *dnaJ*, *dnaK*, *gyrB*, *recA*, or *rpoB* can be used in single-gene-based sieving or multiple-gene-based sieving such as multilocus sequence typing [13] and multilocus sequence analysis [14]. Among them, 16S rRNA, one of the most widely-used marker genes used as the first-line tool for species delineation, has been proposed as an indicator for species delineation [47]. Goris et al. [5] used > 94% 16S rRNA gene sequence identity to sieve closely related pairs for subsequent alignment and calculation. However, our results indicate that FRAGTE is superior to alignment-based approaches due to several reasons. First, some maker genes may have multiple copies in a single genome, which may cause complexity for inferring species relationship especially when the intragenomic diversity is large. For example, *Desulfitobacterium hafniense* carries multiple copies for 16S rDNA gene with intragenomic diversity of up to ~ 5% [12]. Second, the alignment-based approaches are dependent on genomic completeness to identify marker genes, causing the alignment-based approaches to be completeness-dependent. Our testing from the 1779 queries and 264 references showed that 16S rRNA predication depended on genomic completeness (Additional file 1: Figure S9A). Third, some marker genes are lost or challenging to be identified if their sequences are atypical, even when their genomes are complete. For example, the complete genome of *Legionella pneumophila subsp. pneumophila* str. Thunder Bay has only one partial (~ 59%) 16S rRNA identified by the software Barrnap. Fourth, the alignment-based approaches on the basis of sequence identity at the gene level have lower resolutions than our FRAGTE approach. Our testing showed that the 16S rRNA-based approach only sieved the maximal total of 1778 intraspecies pairs even when the cutoff for 16S rRNA identity was set at 84%, as the 16S rRNA identity for *Bifidobacterium longum* subsp. *longum* strain JCM 1217 and strain CCUG30698 was ~ 84% (Additional file 1: Table S1). The number of intraspecies pairs sieved by the 16S rRNA approach was even lower when the cutoff for 16S rRNA identity is set at > 84% (Additional file 1: Figure S9B). In contrast, FRAGTE sieved all 1779 intraspecies pairs. In addition, FRAGTE sieved a total of 7239 pairs (Fig. 5a), while 16S rRNA-based approach needed to sieve a total of 63,024 pairs to achieve the sensitivity similar to FRAGTE (Additional file 1: Figure S9C). Taken together, FRAGTE has higher resolution than the 16S rRNA-based method. Fifth, the relationship indicated by a single or multiple genes may be distorted by HGT, which has litter or almost no effect on FRAGTE, because FRAGTE uses genome-level information instead. Therefore, FRAGTE is more reliable than the alignment-based approaches. Finally, species relationship inferred by multiple marker genes may be inconsistent as they might be under different evolutionary stresses. Also, species relationship inferred by maker gene(s) may be inconsistent to the true species relationship. In contrast, FRAGTE reflects the species relatedness more precisely, as FRAGTE uses the genome-level information to cover a multitude of genes, whose different evolutionary forces are thereby approximately cancelled out.

### FRAGTE cannot completely substitute AF to guarantee high accuracy for species delineation by the ANI approach

Bacteria can take up foreign DNA from the environment though horizontal gene transfer (HGT). Thus, even distantly related species may show a high ANI of > 96%. In this context, delineating such species based only on the ANI criterion may lead to incorrect conclusions. To guarantee high accuracy, AF is usually used together with ANI. For example, MiSI uses a combination of AF and ANI for species delineation [10]. As composition are species specific [15–19], the recipient genome of HGTs may show distinct composition from its donor genome [23]. Thus, we reasoned that FRAGTE could substitute AF to guarantee the high accuracy for the ANI approach by selecting only closely related genomes with similar composition. To explore this hypothesis, we calculated pairwise ANIs and AFs for all pairs from the 1779 queries against 264 references using the MUMmer algorithm (version 3.23) [48]. We found that most pairs with > 96% of ANIs had > 70% of AFs (Additional file 1: Figure S10A). As intraspecies strains have > 70% of AFs [7], there is no problem to delineate them as from the same species. However, around 0.33% (1540/469,656) of pairs with > 96% ANIs had precisely <~ 25% of AFs (Additional file 4). Among them, 95.19% (1466/1540) had < 1% of AFs, possibly owing to the occurred HGT events between them (Additional file 1: Figure S10A). When we used FRAGTE to sieve closely related genomes, we found that FRAGTE pruned all 1447 pairs with putative HGTs (Additional file 1: Figure S10B). Besides, 74 other distantly related pairs with > 96% ANI but <~ 25% AF, possibly owing to contaminations, were also excluded by FRAGTE. It seemed that FRAGTE can substitute AF to guarantee high accuracy for species delineation. However, FRAGTE cannot always guarantee to exclude all distantly related pairs with HGTs or contaminations, especially when the recipient and donor genomes have a similar composition. For example, *Streptococcus pneumoniae* 46 and *Megamonas rupellensis* DSM 19944, which are from different classes (*Bacilli* and *Negativicutes* respectively) to have 99.29% ANI but ~ 0% AF, were sieved by FRAGTE, indicating that FRAGTE cannot completely substitute AF. Therefore, AF must be taken into account to guarantee high species-delineation accuracy even for pairs sieved by FRAGTE.

### Possible application of the FRAGTE approach

Due to its high sensitivity, high specificity, highly reduced number of sieved genomes and highly improved runtime for sieving closely related genomic pairs, all genome-based species-delineation approaches, including ANI [4–6], average amino-acid identity [8, 9] and MiSI [10], and even some multiple-gene-based approaches such as the species identification tool using 40 universal, single-copy phylogenetic marker genes [12], may benefit from FRAGTE to improve their efficiencies. Notably, our FRAGTE approach is modular and can be easily integrated into these species-delineation tools. We anticipant that it will replace TETRA to improve computational efficiency.

Besides, it should be stressed that although FRAGTE was devised to sieve closely related pairs for species delineation, its methodology is general and could readily employed to other bioinformatical tasks. For example, as fragment composition is widely used to classify metagenomic assemblies into species-related units, FRAGTE may be useful for binning, especially when merging sub-bins. Thus, we plan to integrate FRAGTE into binning approaches in the future.

## Conclusion

Here, we present a novel sieving approach termed FRAGTE. We demonstrate that FRAGTE is completeness-independent and is able to sieve closely related pairs with high sensitivity (~ 100%) as well as high specificity. In addition, we demonstrate that our method runs faster than TETRA to reduce computing cost for the sieving process and sieves a lower total number of genomes for subsequent alignment and calculation to reduce computing cost for the process after sieving, thereby together reducing the computing cost for species delineation. Besides, we demonstrate that FRAGTE is unable to completely substitute AF to guarantee high species-delineation accuracy for the ANI approach. Therefore, we anticipant that FRAGTE will be coupled with modern tools to facilitate taxonomic studies at the species level and also further developed for other application for prokaryotes.

## Methods

### Genome selection

All 6230 complete genomes were downloaded from the NCBI database (on 20 January 2017). To evaluate and compare the sieving performance, we chose genomes with unambiguous species affiliation. Based on the List of Prokaryotic names with Standing in Nomenclature (LPSN) database [49], 5139 genomes with validated species taxa were selected. Then, genomes belonging to species with only one sequenced strain were discarded. We obtained 3953 genomes belonging to 458 species. For each species, we selected a representative genome as the reference. Genomes belonging to type strains, which were recognized using the Straininfo bioportal [50] and the LPSN database, were selected as references. However, in the absence of a type strain, the genome with the largest genome size was selected as the reference. The remaining genomes were used as queries. Finally, 1779 queries and 264 references listed in https://github.com/Yizhuangzhou/FRAGTE were selected for this study.

### MAG download and selection

All 10,445 MAGs were downloaded from the NCBI database specialized for metagenomes (ftp://ftp.ncbi.nlm.nih.gov/genomes/genbank/metagenomes/) on 20 November 2019. Then we filtered MAGs by three steps. First, 194 MAGs with genome size < 10 kb were filtered, as only inter- or intra-species genomes with size > 10 kb can be well separated (Fig. 3a). Second, as most of genomes are < 10 Megabase pair (Mb) (Additional file 1: Figure S11), 6895 MAGs with size > 10 Mb, which may have some contamination, were filtered. Finally, 334 genomes were filtered due to having no counterparts (TETRA > 0.8), as intraspecies genomes with only ~ 10% completeness have a TETRA > 0.8 (Fig. 1). After filtering, 3032 MAGs were remained and compared in a pairwise manner by using the MUMmer algorithm (version 3.23) according to the method in [4]. 94,618 pairs with an ANI > 96% and an AF > 60% were considered as intraspecies, according to the MiSI method [10].

### The calculation of TETRA

Ambiguous nucleotides (that is not A, T, C, or G) within any genome were discarded. The processed genomes were then concatenated with their reverse complements and the tetranucleotide frequencies were computed. These frequencies were subsequently transformed into z-scores following the method of Teeling et al. [19]. The TETRA (similarity) between two genomes was calculated as the PCCD for their tetranucleotide frequency-derived z-scores.

### Fragmenting phase in FRAGTE

Genomes (> 10 kb) were remained for further processing. For each genome, all contigs/scaffolds were concatenated. Next, the concatenated genomes were divided using a sliding window of *l* kb (with 0.5 *l* kb overlap). A genome was required to divide into ≥8 fragments with length in the range of 10–200 kb by setting *l* as *L*/4, given that the total size of the concatenated genome was denoted *L*. As 4 is the minimal number for calculating a creditable GSC, setting *l* as *L*/4 ensures that fragments are as long as possible to increase their specificity. If *L* is < 40 kb, no fragmenting was performed for this genome and LSC with length of the entire genome was used as GSC (Additional file 1: Figure S5). If *L* was within the range of 40 kb to 800 kb, the genome was divided into 8 fragments by setting *l* to *L*/4 and a GSC can be calculated based on the divided fragments. If *L* was > 800 kb, the genome was divided into ≥8 fragments by compelling FRAGTE to set *l* to the maximally allowed 200 kb, which increased the sampling number (fragment number) to possibly generate a small GSC to avoid missing some intraspecies pairs. This design is very rational and useful, as the GSCs increased with fragment size due to increased mean intragenomic PCCD but decreased SD (Fig. 3b). A GSC can also be calculated based on its divided fragments.

After genome fragmenting, all fragments were subject to calculation of z-scores according to the method of Teeling et al. [19] (Fig. 4a). Subsequently, for each fragment, PCCDs were calculated with all non-overlapped intragenomic fragments (see section on “The calculation of TETRA”). The fragment with the maximal accumulated PCCD was selected to represent its genome. In addition, a fourfold longer fragment consisting of 4 fragments with top 4 largest accumulated PCCDs was were also subject to calculation of z-scores according to the method of Teeling et al. [19]. Finally, FRAGTE calculated the mean (*Mean*) and *SD* of all PCCDs for the representative fragments to compute a GSC for its genome as follows:

$$ GSC=M\mathrm{ean}-2\ast SD $$

Then, if the LSC corrosponding to the size (kb) of the representative fragment was denoted LSC_kb_, we restricted the GSC as follows:

$$ GSC=\Big\{{\displaystyle \begin{array}{c}{\mathrm{LSC}}_{\mathrm{kb}},\mathrm{if}\ \mathrm{GSC}<{\mathrm{LSC}}_{\mathrm{kb}}\\ {}0.92,\mathrm{if}\ \mathrm{GSC}>0.92\end{array}} $$

As GSCs are used for genome filtering, GSCs should be equal to or larger than LSC_kb_. Besides, we found that the cutoff, which is calculated as the mean intragenomic PCCD minus one SD, increases with fragment size to obtain a maixmal value of 0.92 (Additional file 1: Figure S12A). Using 0.92 as GSC filters ~ 100% of interspecies pairs (Additional file 1: Figure S12B), according to the empirically-determined PCCD distributions (Fig. 3b and Additional file 3). This indicates that using a > 0.92 GSC may not greatly increase FRAGTE specificity. Thus, to avioid to filter some intraspecies pairs due to the larger GSCs, we restricted the maximally allowed GSC as 0.92.

Through the fragmenting phase, FRAGTE obtained 256 z-scores for the representative fragment (ZRF) and the fourfold longer fragment (ZLF) as well as one GSC for each genome. Selecting by LSC requires that the fragment within the range of 10–200 kb. If *L* is ≤200 kb, FRAGTE considers ZLF as ZRF to improve selecting sensitivity, since selecting sensitivity increases with fragment size (Additional file 1: Figure S5).

### Determining phase in FRAGTE

For a given pair, FRAGTE obtains a couple of ZRFs, ZLFs and GSCs for its query and reference in its fragmenting phase. In the determining phase, FRAGTE calculates two PCCDs for each pair, one for a pair of ZRFs (termed P1) and the other for a pair of ZLFs (termed P2) (Fig. 4b). If P1 is ≥ LSC, FRAGTE selects this pair. Then the selected pair is subject to filtering by GSC. The smaller GSC between the query’s GSC and the reference’s GSC is taken as the GSC of this pair (term GSC_p_). If P1 is ≥GSC_p_, this pair is finally sieved as a closely related pair. Otherwise, this pair is subject to comparing with P2. If P2 is ≥GSC_p_, the pair is also finally sieved as a closely related pair.

### 16S rRNA analysis

16S rRNA genes were predicted by the software Barrnap (BAsic Rapid Ribosomal RNA Predictor, version 0.7, https://github.com/tseemann/barrnap/releases/). Pairwise identities between 16S rRNA genes were calculated based on global alignment tool CLUSTAL W (version 2.0.12) [51]. To ensure the reproducibility of the similarity calculation, we used CLUSTAL W to align only two sequences at a time. Pairwise similarities were then calculated without considering alignment gaps, following the suggestion of Chun et al. [52].

## Supplementary information

**Additional file 1: Figure S1.** This diagram illustrates the sieving process and its contribution to species delineation by whole-genome approaches. **Figure S2.** Illustration of genome-specific composition. **Figure S3.** Determination and sensitivity of the length-specific cutoffs (LSCs). **Figure S4.** Genome-specific cutoffs (GSCs). **Figure S5.** Setting of *l* in FRAGTE. **Figure S6.** Statistics for both Genomes and MAGs. **Figure S7.** Sieving performance comparison of FRAGTE and TETRA on MAGs. **Figure S8.** Sieving performance of FastANI on simulated genomes. **Figure S9.** Sieving performance of the 16S rRNA-based approach. **Figure S10.** FRAGTE pruned pairs with > 96% ANI but <~ 25% AF. **Figure S11.** Genome size distributions for both genomes and MAGs. **Figure S12.** Detemination and specificity of the maximally allowed GSC. **Table S1.** Pairwise alignments between 16S rRNAs in *Bifidobacterium longum* subsp. *longum* strains JCM 1217 and CCUG30698.

**Additional file 2: Table S1.** All 1779 complete query genomes. **Table S2.** All 264 complete reference genomes. **Table S3.** All 61,914 queries of real genomes. **Table S4.** All 5680 references of real genomes. **Table S5.** 3032 MAGs.

**Additional file 3.** Empirically-determined PCCD distributions and the corresponding length-specific cutoffs (LSCs) used in this study.

**Additional file 4.** 1540 pairs with ANIs > 96% but AFs < ~ 25%.

## Data Availability

All data generated or analyzed during this study are included supplementary information files (see tables in Additional file 2). Additionally, the TZMD approach and all tested data are available at https://github.com/Yizhuangzhou/FRAGTE.

## Abbreviations

AF: Alignment fraction
ANI: Average Nucleotide Identity
FRAGTE: Fragment tetranucleotide frequency correlation coefficient
GSC: Genome-specific cutoff
HGT: Horizontal gene transfer
kb: kilobase pair
LPSN: List of Prokaryotic names with Standing in Nomenclature
LSC: Length-specific cutoff
MAG: Metagenome-assembled genome
Mb: Megabase pair
MiSI: Microbial Species Identifier
NCBI: National Center for Biotechnology Information
PCCD: Pearson correlation coefficient distance
SD: Standard deviation
SMRT: Single-molecule, real-time
TETRA: Genome-wide tetranucleotide frequency correlation coefficient
TZMD: Tetranucleotide-derived Z-value Manhattan Distance
ZLR: Z-scores for the fourfold longer fagment
ZRF: Z-scores for the representative fragment
