# Supplementary material for: A completeness-independent method for pre-selection of closely related genomes for species delineation in prokaryotes
Source: BMC Genomics. 2020 Feb 26;21:183. doi: 10.1186/s12864-020-6597-x (PMC7045542; doi:10.1186/s12864-020-6597-x)
Supplement: Supplementary file 1 — Additional file 1: Figure S1. This diagram illustrates the sieving process and its contribution to species delineation by whole-genome approaches. Figure S2. Illustration of genome-specific composition. Figure S3. Determination and sensitivity of the length-specific cutoffs (LSCs). Figure S4. Genome-specific cutoffs (GSCs). Figure S5. Setting of l in FRAGTE. Figure S6. Statistics for both Genomes and MAGs. Figure S7. Sieving performance comparison of FRAGTE and TETRA on MAGs. Figure S8. Sieving performance of FastANI on simulated genomes. Figure S9. Sieving performance of the 16S rRNA-based approach. Figure S10. FRAGTE pruned pairs with > 96% ANI but <~ 25% AF. Figure S11. Genome size distributions for both genomes and MAGs. Figure S12. Detemination and specificity of the maximally allowed GSC. Table S1. Pairwise alignments between 16S rRNAs in Bifidobacterium longum subsp. longum strains JCM 1217 and CCUG30698. [file 12864_2020_6597_MOESM1_ESM.pdf]

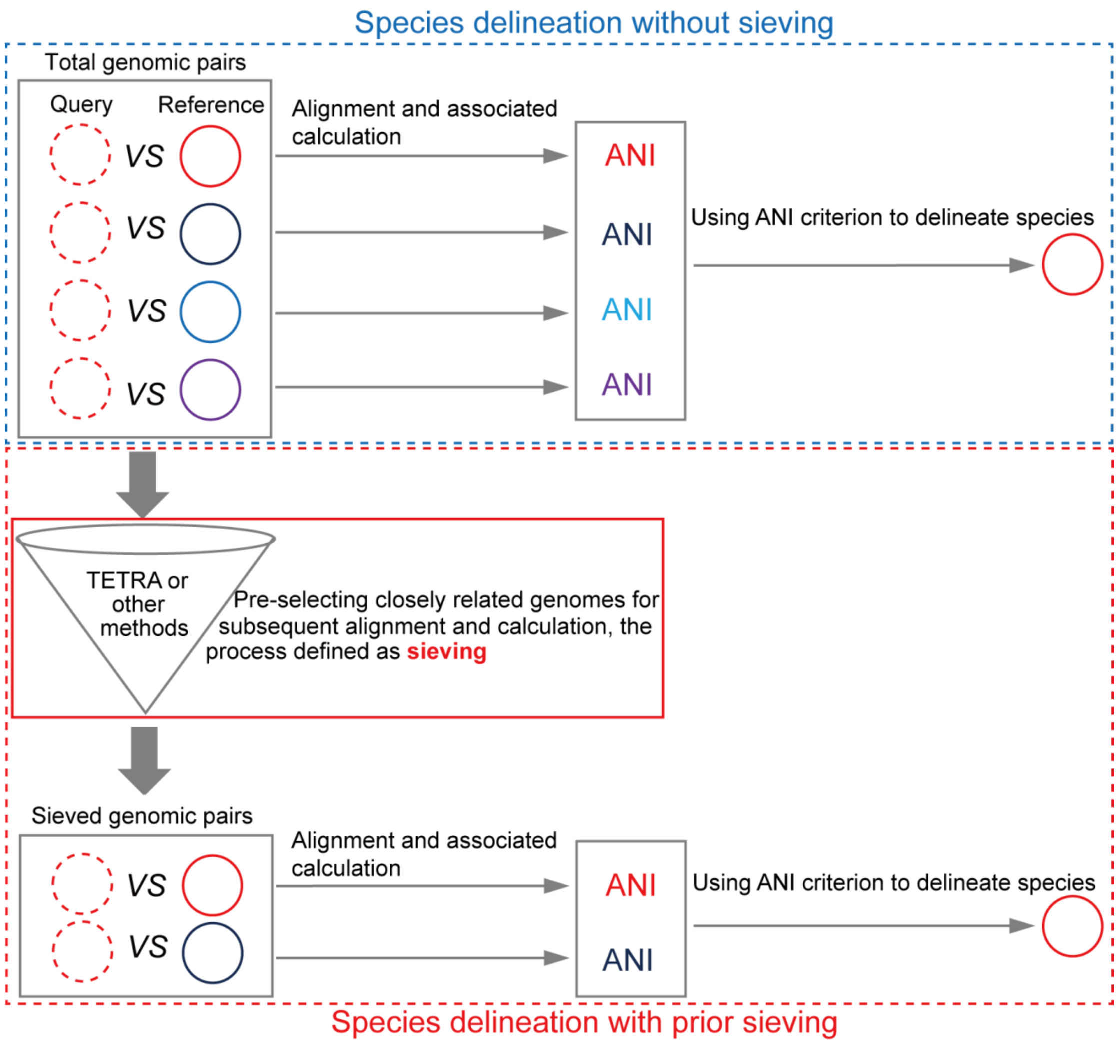


Figure S1. This diagram illustrates the sieving process and its contribution to species delineation by whole-genome approaches. Sieving is defined as the process to pre-select closely related genomes for subsequent alignment and calculation (boxed in a solid red border). For example, given one intraspecific and three interspecific genomic pairs (one query against four references), without pre-selection by the sieving algorithm, the ANI approach would require four pairwise alignments and ANI calculations. Alternatively, the ANI approach only needs two alignments and associated calculations after sieving. Therefore, sieving reduces the total computational cost (fewer pairs for alignment and associated calculation). It is important to emphasize that sieving does not directly delineate species, as some interspecific pairs are still able to perforate the mesh of the sieving algorithm.

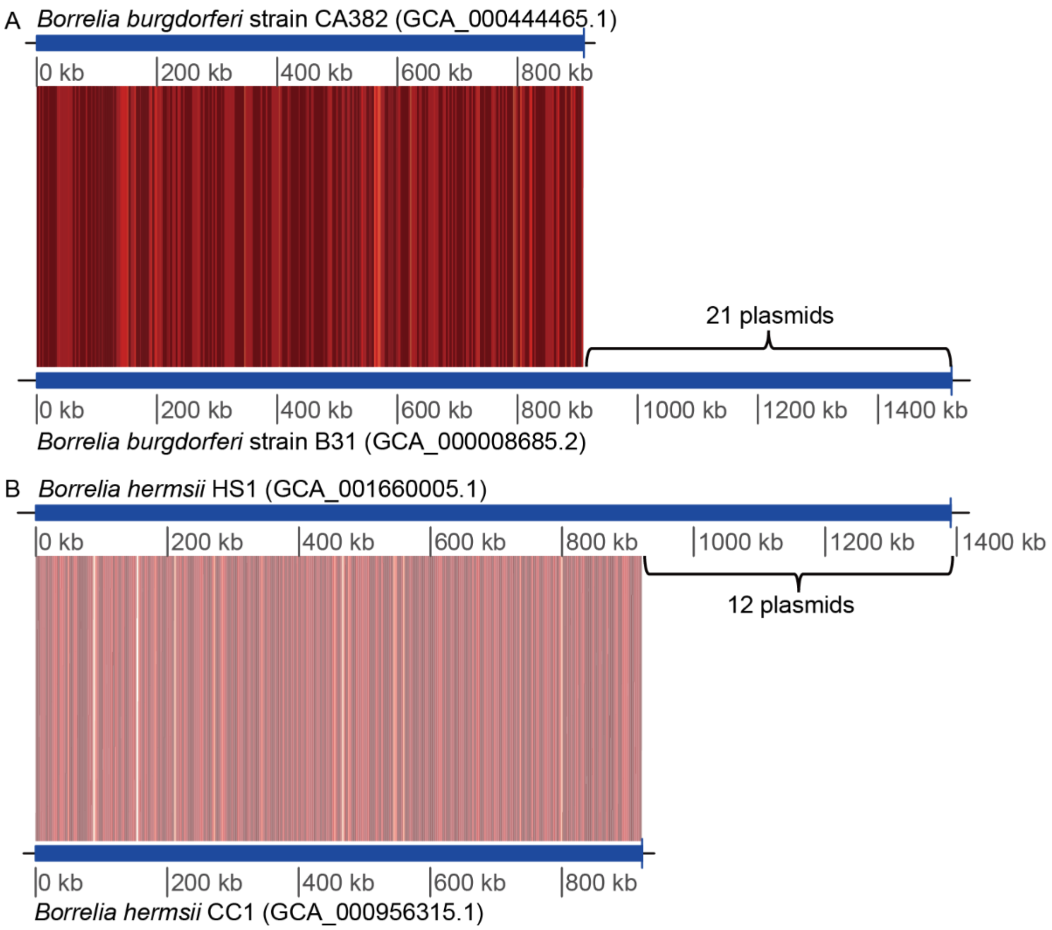


Figure S2. Illustration of genome-specific composition. A, for *Borreliella burgdorferi* strain CA382 and strain B31, showing that the composition difference is mainly derived from plasmid difference; B, *Borrelia hermsii* strain HS1 and strain CC1, also showing that the composition difference is mainly derived from plasmid difference. Line segments denote the orthologous mappings.

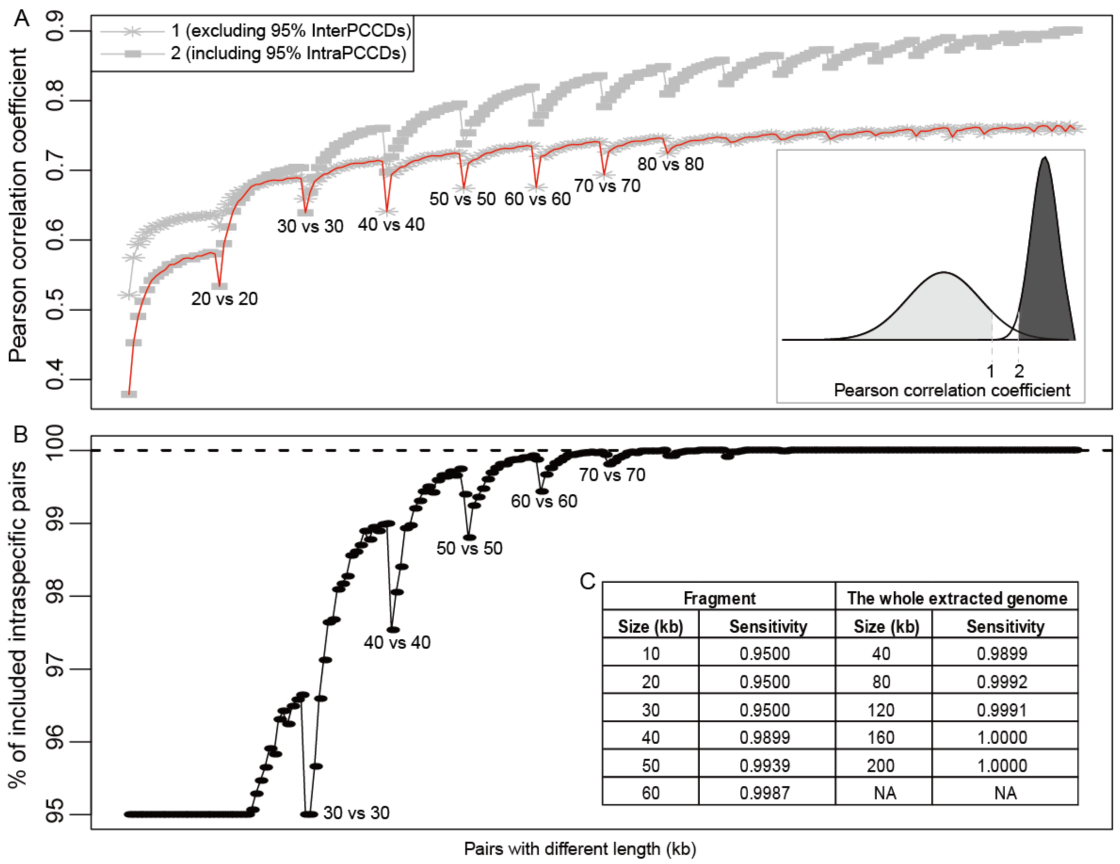


| Fragment | | The whole extracted genome | |
|---|---|---|---|
| Size (kb) | Sensitivity | Size (kb) | Sensitivity |
| 10 | 0.9500 | 40 | 0.9899 |
| 20 | 0.9500 | 80 | 0.9992 |
| 30 | 0.9500 | 120 | 0.9991 |
| 40 | 0.9899 | 160 | 1.0000 |
| 50 | 0.9939 | 200 | 1.0000 |
| 60 | 0.9987 | NA | NA |

Figure S3. Determination and sensitivity of the length-specific cutoffs (LSCs). A, determination of the LSCs. *Inset*, showing the cutoff points to exclude 95% of interspecific PCCDs (InterPCCDs) and include 95% of intraspecific PCCDs (IntraPCCDs); B, sensitivity to include intraspecific pairs; C, Increased sensitivity for small-sized fragments (<60 kb) by using the entire extracted genome rather than the divided fragment for selecting by LSC. For pairwise sizes in the x axis, please refer to Additional file 2: Table S1. All were based on empirically-determined PCCD distributions (Fig. 3B and Additional file 2: Table S1). NA, not applicable, as the maximal allowed size for LSC is 200 kb but the size of entire extracted genome is >240 kb.

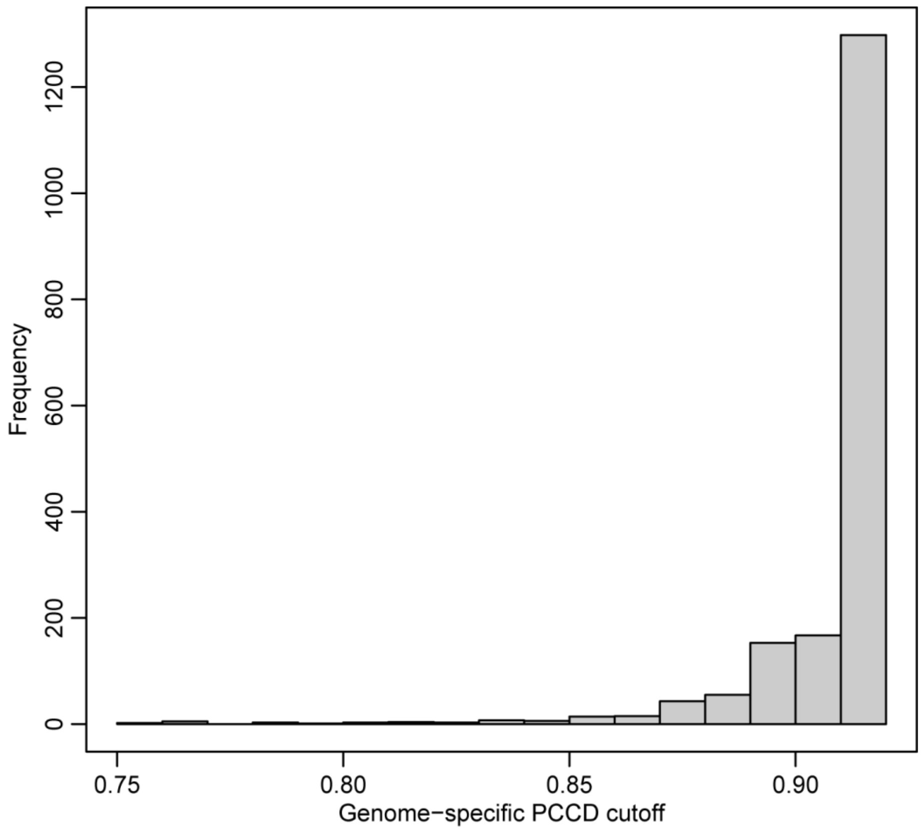


Figure S4. Genome-specific cutoffs (GSCs). This example was produced with the summary of 1,779 query genomes with 60% completeness. The GSC was calculated as the mean PCCD minus two standard deviations with two restrictions (for details, see Materials and Methods).

A

```
# L in base pair (bp)
if (L < 40,000) {
   # short genomes
   kb = int (L/10,000)*10;
   GSC = LSCkb
}  # LSCkb: LSC when size is kb
else { # long genomes
   l = L / 4;
   if (l > 200,000) {
     l = 200,000;
   }
}
```

B

| ***L* (kb)** | ***l* (kb)** | # of fragments | GSC |
|---|---|---|---|
| [10,40) | NA | NA | LSC |
| [40,800] | *L* /4 | 8 | GSC |
| >800 | 200 | ≥8 | GSC |

Figure S5. Setting of *l* in FRAGTE. A, the pseudo code for setting *l*; B, summary when *L* is different. *L*, the length of a genome (kb); NA, not applicable, represents no fragmenting; GSC, genome-specific cutoff; LSG, length-specific cutoff.

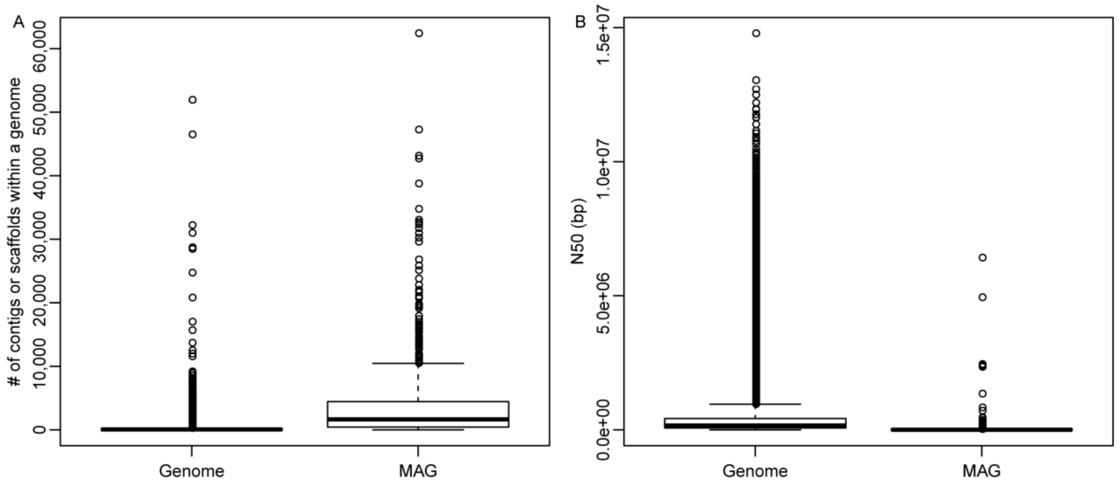


Figure S6. Statistics for both genomes and MAGs. A, for number of contigs or scaffolds within a genome; B for N50. N50, a length (bp) for which the collection of all contigs/scafolds of that length or longer contain at least 50% of total size of its genome/MAG. 3032 MAGs and 83,061 genomes were used here.

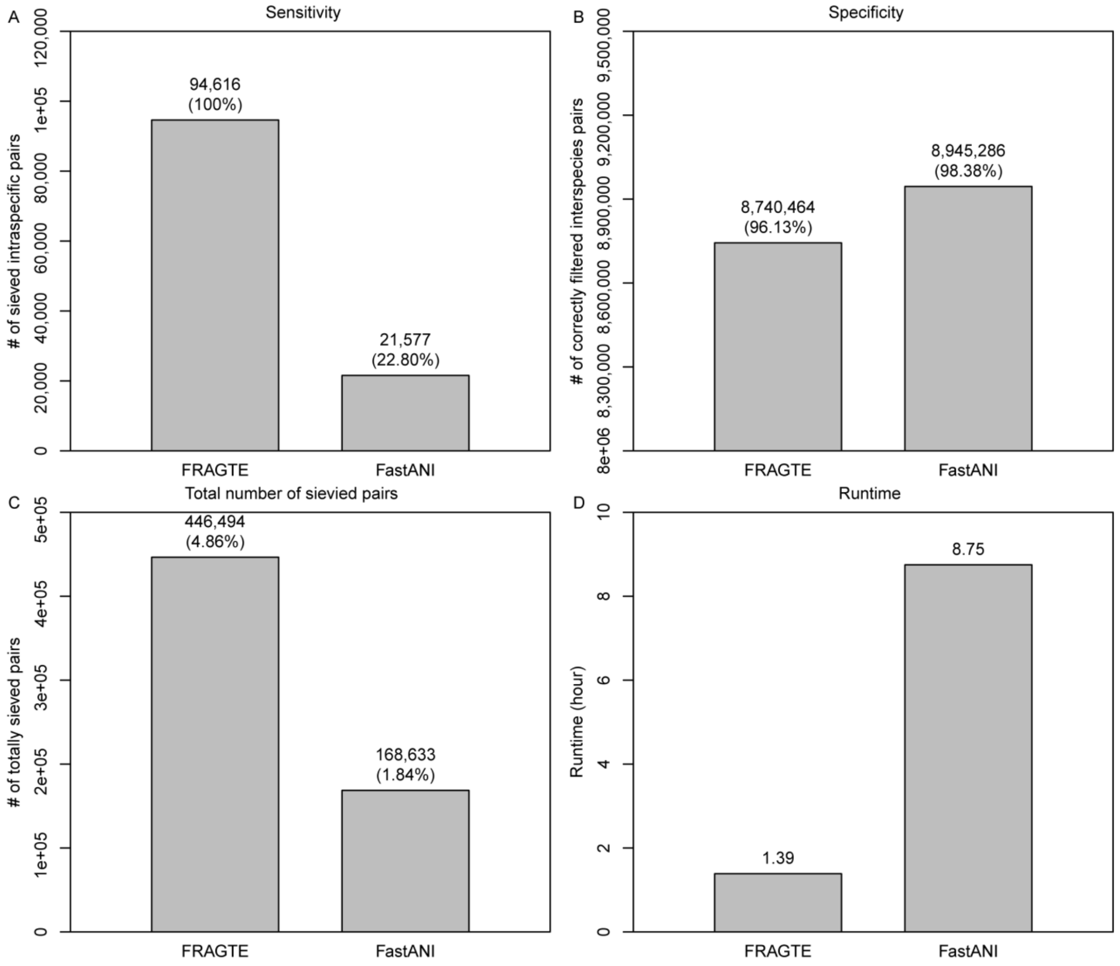


Figure S7. Sieving performance comparison of FRAGTE and TETRA on MAGs. A, for sensitivity; B, for specificity; C, for total number of sievied pairs; D, for runtime. The runtime is the summed executive time including both fragmenting and determining phrases for all pairs by using serial execution (single thread, single process). Only a single compute node with two Intel® Xeon® Silver 4114 20-core processors was used. All were run on 3032 MAGs (Additional file 2: Table S5) against themselves, which comprise 94,618 intra- and 9,095,374 inter-species pairs.

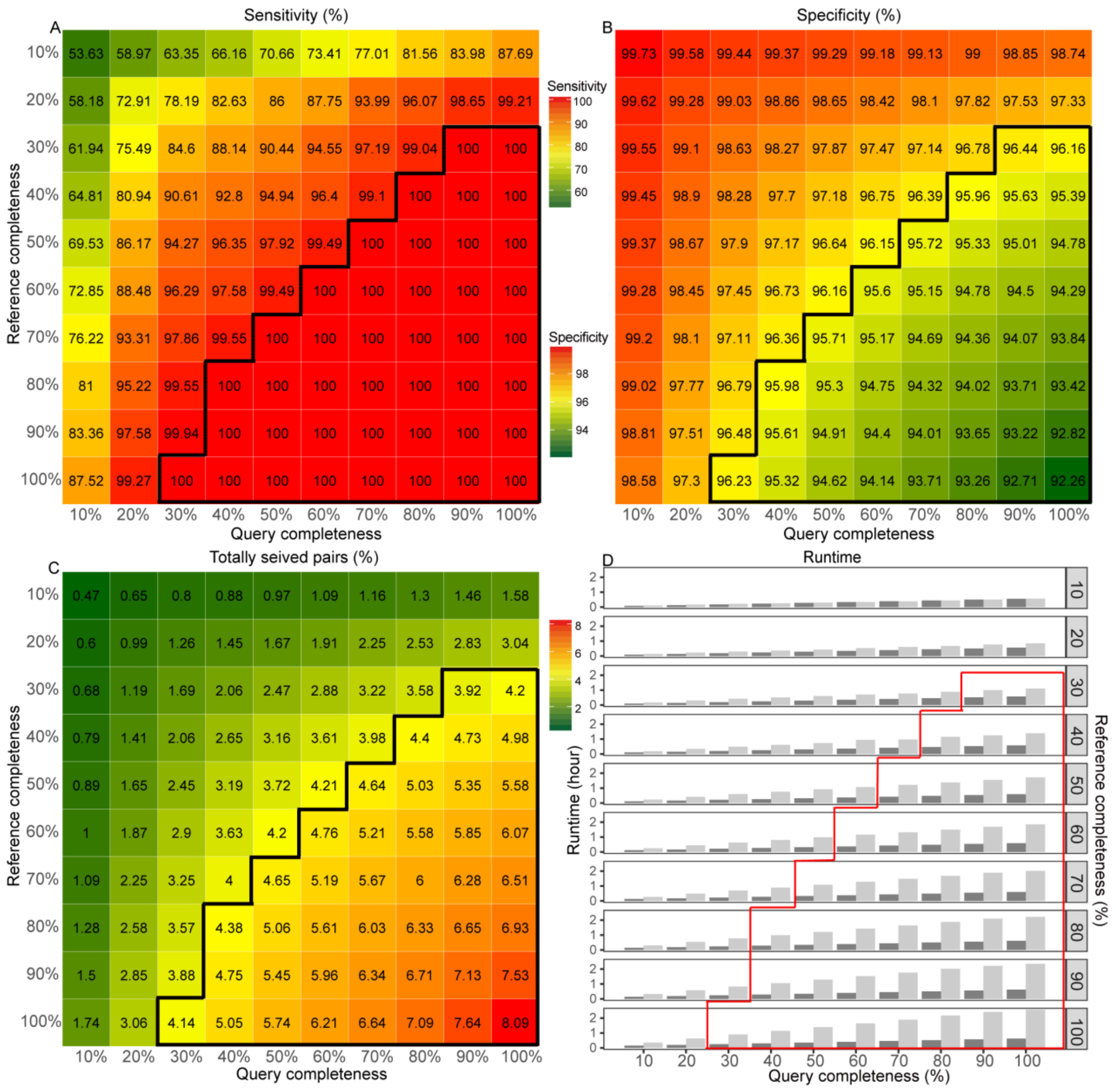


Figure S8. Sieving performance of FastANI on simulated genomes. A, for sensitivity; B, for specificity; C, for percentage of totally sieved pairs, which is calculated as total number of sieved pairs divided by the total number of pairs; D, for runtime. The number in cell is used as a basis for color intensity. All were run on 1779 queries (Additional file 2: Table S1) against 264 references (Additional file 2: Table S2). Solid box, sieving with the same sensitivity (100%) as FRAGTE.

.

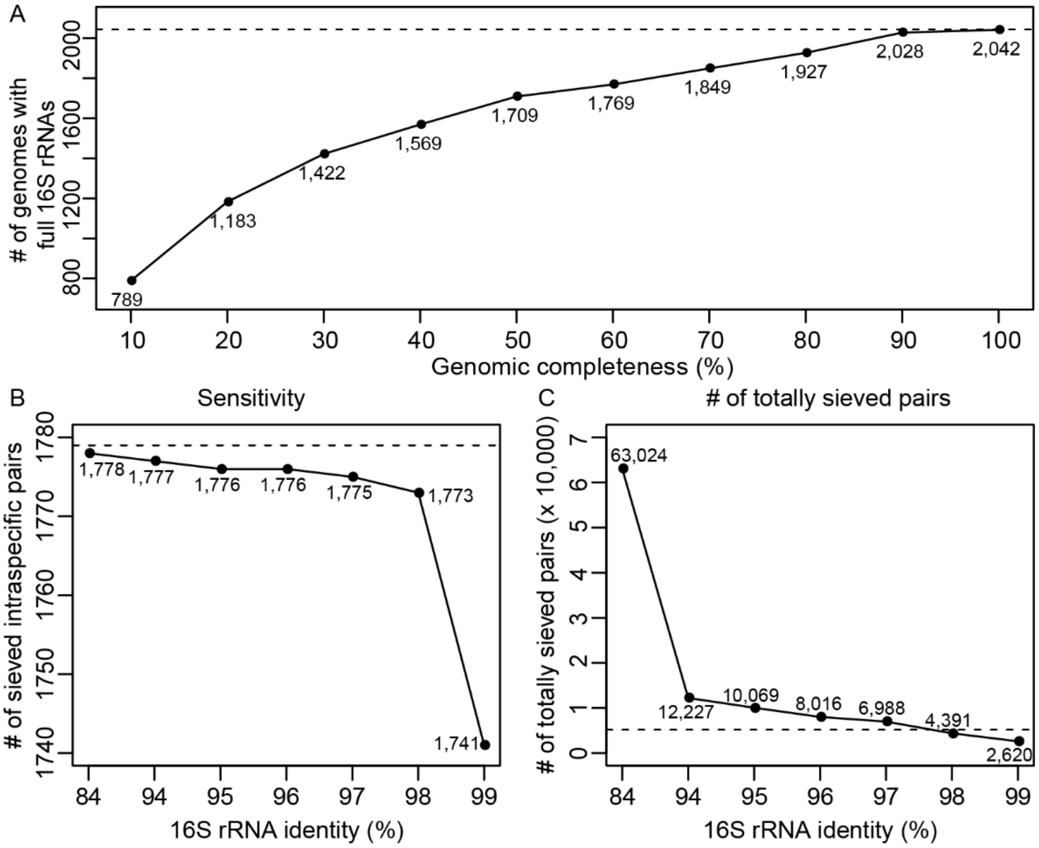


Figure S9. Sieving performance of the 16S rRNA-based approach. A, identification of 16S rRNAs is dependent on genomic completeness; B, sieving sensitivity of the 16S rRNA-based approach, given that all tested genomes are complete; C, number of totally sieved pairs by 16S rRNA-based approach, given that all tested genomes are complete. All were from the 1,779 queries (Additional file 2: Table S1) against 264 references (Additional file 2: Table S2).

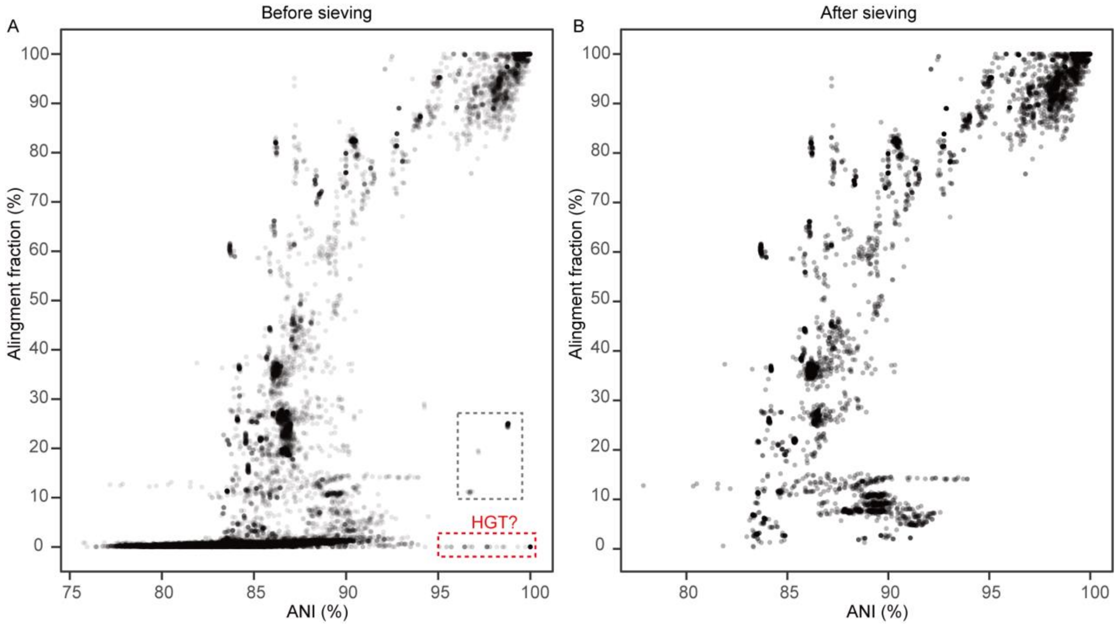


Figure S10. FRAGTE pruned pairs with >96% ANI but <~25% AF. A, before sieving. Dashed red box, pairs with putative horizontal gene transfer (HGT) events; Dashed grey box, pairs with an ANI of >96% but a AF <~25% possibly due to contamination; only pairs with an ANI of >75% are shown; to reduce memory size, a randomly-selected 1% of points for pairs with ANI <10% are shown. B, after sieving by FRAGTE.

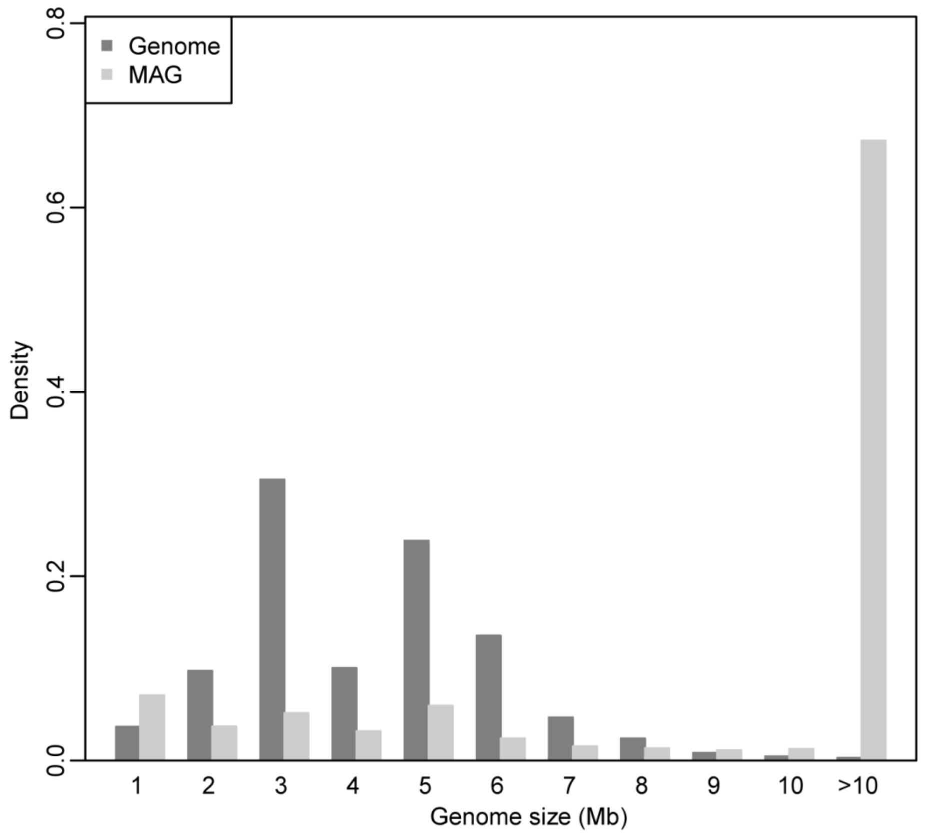


Figure S11. Genome size distributions for both genomes and MAGs. 83,060 genomes and 10,252 MAGs with size >10 Kb were used. All genomes and MAGs were downloaded from the NCBI database. Mb, megabase pair.

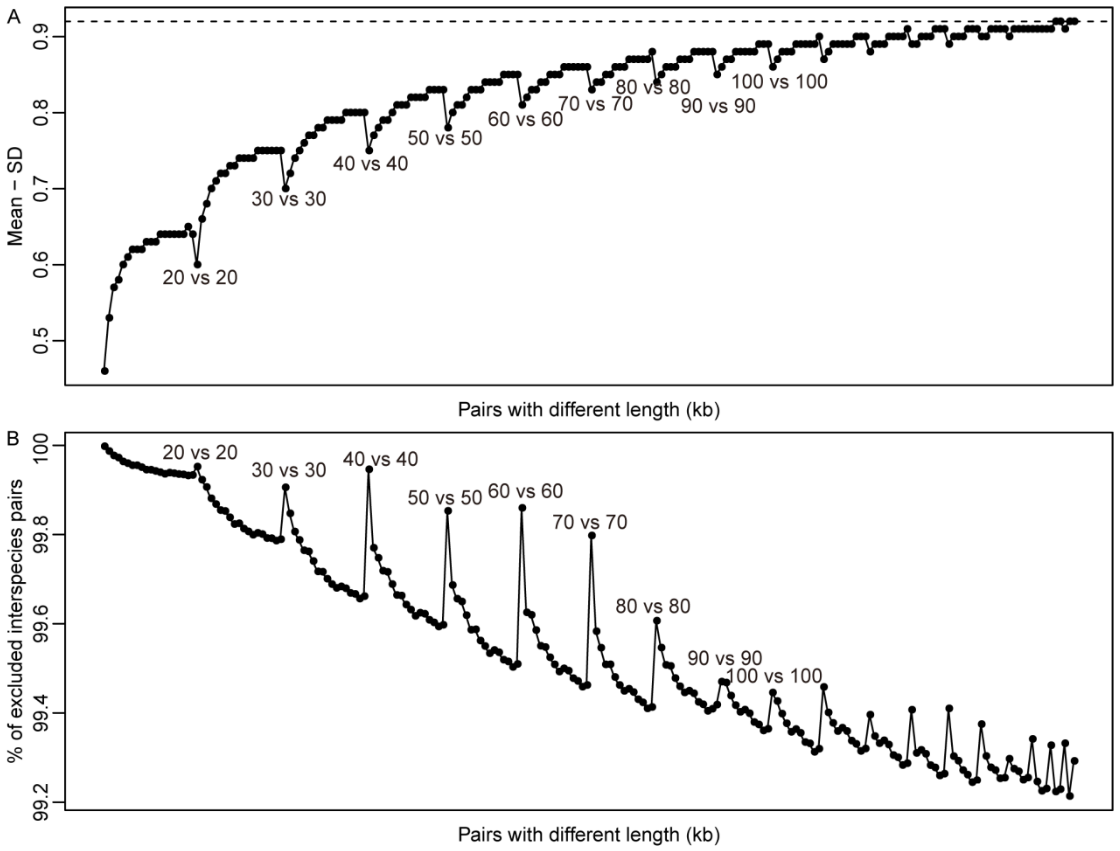


Figure S12. Detemination and specificity of the maximally allowed GSC. A, determination of the maximally allowed GSC as 0.92; B, specificity of using 0.92 as the maximally allowed GSC. For pairwise sizes in the x axis, please refer to Additional file 3. All were based on empirically-determined PCCD distributions (Fig. 3B and Additional file 3). Mean and standard deviation (SD) represent the average and SD of the intraspecies PCCD distribution.

Table S1. Pairwise alignments between 16S rRNAs in *Bifidobacterium longum* subsp. *longum* strains JCM 1217 and CCUG30698.

| 16S rRNA in GCA_000196555.1 | 16S rRNA in GCA_001446275.1 | Identity (%) |
|---|---|---|
| CP011965.1\|1851194-1852708\|+[$] | AP010888.1\|2294150-2295671\|- | 84.2 |
| CP011965.1\|1851194-1852708\|+ | AP010888.1\|2287973-2289494\|- | 84.2 |
| CP011965.1\|1851194-1852708\|+ | AP010888.1\|2082553-2084074\|- | 84.27 |
| CP011965.1\|1851194-1852708\|+ | AP010888.1\|1538331-1539852\|- | 84.2 |
| CP011965.1\|1652630-1654139\|- | AP010888.1\|2294150-2295671\|- | 84.88 |
| CP011965.1\|1652630-1654139\|- | AP010888.1\|2287973-2289494\|- | 84.88 |
| CP011965.1\|1652630-1654139\|- | AP010888.1\|2082553-2084074\|- | 84.95 |
| CP011965.1\|1652630-1654139\|- | AP010888.1\|1538331-1539852\|- | 84.88 |

Note: [$] "CP011965.1" is the scaffold ID; "1851194" is the start position; "1852708" is the end position; "+" indicates the DNA strand. Bifidobacterium longum subsp. longum strain JCM 1217 (GCA_000196555.1) and stain CCUG30698 (GCA_001446275.1).
